# Accurate Simulations of Water and Aqueous Solutions through Fine-Tuned Dispersion-Corrected Density Functional Theory and Machine-Learning Interatomic Potentials

**DOI:** 10.1021/acs.jcim.5c02079

**Published:** 2025-11-12

**Authors:** Alfonso Ferretti, Giacomo Melani, Luca Benedetti, Robert A. Sorodoc, Alessando Fortunelli, Giuseppe Brancato

**Affiliations:** † 19004 Scuola Normale Superiore, Piazza dei Cavalieri 7, I-56127 Pisa, Italy; ‡ Istituto Nazionale di Fisica Nucleare (INFN), Sezione di Pisa, Largo Pontecorvo 3, I-56127 Pisa, Italy; § Consiglio Nazionale delle Ricerche, CNR-ICCOM, 56124 Pisa, Italy; ∥ NEST Istituto Nanoscienze-CNR and Scuola Normale Superiore, 56127 Pisa, Italy; ⊥ Scuola Normale Superiore and CSGI, Piazza dei Cavalieri 7, I-56127 Pisa, Italy

## Abstract

Dispersion-corrected density functional theory (DFT-D) is widely employed to model large molecular systems at an affordable computational cost and to develop machine-learning interatomic potentials (MLIPs), enabling reliable molecular dynamics (MD) simulations of condensed-phase systems. Yet, given a molecular system, the choice of a specific DFT-D model that can achieve the necessary accuracy over an extended range of physicochemical properties and conditions is generally not trivial. Here, we report an effective computational strategy for enhancing the accuracy of standard DFT-D models toward high-level quantum mechanical data and for developing MLIPs preserving the same high fidelity. Taking water as a paradigmatic example, we derive a novel MLIP and demonstrate that its use allows us to accurately predict a wide range of properties in diverse forms, from small clusters to bulk liquid and ice, such as radial distribution functions, fusion/vaporization enthalpies, diffusion constants, and density isobars, capturing remarkably well its peculiar and anomalous behavior, often elusive even to standard first-principle MD simulations. Furthermore, we show how the same computational strategy can be readily extended to treat aqueous solutions. Considering MgCl_2_ in water as a test case, we develop a MLIP and use it to predict the metal ion hydration structure and the water exchange dynamics exhibiting a significantly improved agreement with experiments with respect to both standard DFT-D and classical force fields.

## Graphical abstract

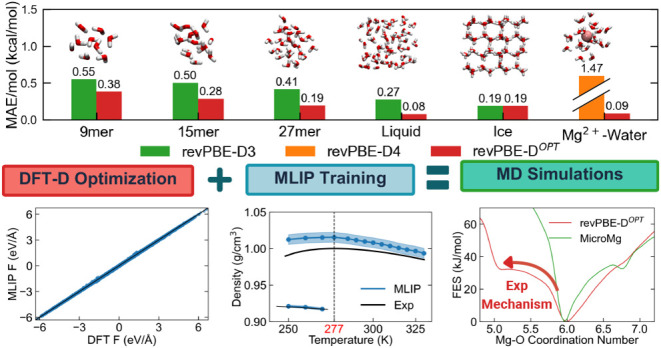

## Introduction

Machine learning interatomic potentials (MLIPs) based on neural networks are becoming the *de facto* approach to reliably simulating condensed-phase molecular systems at an affordable computational cost.^1–3^ However, the quality and performances of MLIPs are mostly determined by two key factors: (1) the ability to correctly infer short-range interactions that implicitly include long-range interactions such as dispersion and electrostatics (since long-range interactions are still difficult to incorporate into MLIPs)^4^ and (2) the generation of large and accurate training databases relative to condensed-phase molecular structures,^5^ especially those representing complex and disordered liquid phases. In this regard, reliable training data for MLIPs can be conveniently obtained through density functional theory (DFT) methods,^6–8^ which generally show a good balance between accuracy and efficiency. Indeed, DFT offers a computationally more favorable scaling with the number of electrons than gold standard quantum mechanical methods (i.e., explicitly correlated coupled cluster or quantum Monte Carlo methods), although approximate exchange-correlation functionals may compromise accuracy, e.g., underestimating van der Waals (VdW) dispersion interactions.^9^ For a given molecular system, it is then usually not trivial to choose a density functional that achieves the needed accuracy over a wide range of physicochemical properties and conditions (from small isolated clusters to the liquid and solid phases, and from local structural properties to basic thermodynamics, such as bulk density, diffusion, enthalpy of melting/vaporization, vibrational density of states, etc.). This is clearly illustrated by the case of water, that is still debated, despite its intrinsic interest justifying the tremendous efforts devoted to its computational description and despite the improvements demonstrated recently by the latest DFT approximations.^8,10–15^ In this context, recent developments in density-corrected SCAN functionals, belonging to the third (meta-GGA) rung of the Jacob’s ladder of DFT approximations, are particularly noteworthy, such as the works of Paesani and co-workers^16–18^ in which applications to pure water systems led to the accurate description of several water properties when combined with a many-body expansion potential.^13^ Nevertheless, weak dispersion forces are generally not well represented by these functionals and further corrections have been proposed.^14^ Among others, two recently proposed approaches are the experiment directed simulations^19^ (EDS) or the “Δ-learning”^20^ used to promote DFT to Coupled-Cluster accuracy. In this context, the addition of semiempirical dispersion corrections to standard Kohn–Sham DFT (DFT-D) models^21^ represents one of the most widely used and well-assessed in silico approaches for treating complex molecular systems because of the demonstrated capability to recover the missing VdW interactions affecting popular exchange-correlation functionals.^22,23^

Recently, we proposed the idea of fine-tuning Grimme’s DFT-D models toward specific molecular systems, so as to elevate the accuracy of the computed noncovalent interactions up to high-level reference calculations (i.e., achieving a mean absolute error of 0.1 kcal/mol per molecule),^24,25^ with the final goal of improving the description of condensed-phase properties. It should be noted, however, that this approach is physically sound and computationally feasible as far as the focus is on standard equilibrium and dynamical properties, while more subtle electronic properties may need different strategies.^26–28^ Accordingly, the DFT-D models were reoptimized by tweaking the empirical parameters of the potential (i.e., scaling factors) to match benchmark interaction energies issuing from hundreds of nonequilibrium molecular configurations. This was achieved by factoring out from the reference calculations the energy deviations of the one-body contributions (i.e., monomer energy deviations), thus refining the dispersion corrections more effectively. Test calculations carried out on acetonitrile^24^ and water^25^ proved this approach successful in obtaining highly accurate DFT-D models with respect to reference diffusion Monte Carlo (DMC),^29^ making it particularly well-suited for large-scale applications, such as *ab initio* molecular dynamics (AIMD) simulations or data generation for MLIPs.

Here, based on our previous work, we applied our DFT-D optimization protocol to water and an aqueous solution of MgCl_2_. Then, we exploited the high-fidelity data generated by the optimized DFT to build effective MLIPs based on the message-passing atomic cluster expansion (MACE) architecture.^30^ Remarkably, the obtained MLIPs are capable of representing liquid water and ice at the level of the best state-of-the-art potentials. Moreover, we demonstrate that the water MLIP is transferable to salt solutions, showing that the MgCl_2_/water MLIP we derive starting from the pure water MLIP is able to correctly represent both the hydration structure and the water exchange mechanisms in the first solvation shell of the magnesium ion in better agreement with experiment than alternative approaches.

## Methods

### DFT-D Optimization

The *rev*PBE-D3 model was reoptimized toward the reference DMC data set of Alfè et al.^12^ on water interaction energies obtained from liquid-phase configurations containing either 32 or 64 water molecules, enforcing periodic boundary conditions. Following the procedure originally presented in refs 24,25 we obtained an optimal parametrization that minimized the mean absolute error (MAE) in the interaction energy relative to diffusion Monte Carlo (DMC) calculations by excluding the one-body energy contribution issuing from individual water molecules using the Partridge and Schwenke potential.^31^ All DFT calculations were carried out using a plane-wave basis set and soft pseudopotentials^32^ as implemented in the Quantum Espresso package.^33^ No significant differences were observed in comparison with all-electron quantum mechanical calculations employing the extended aug-cc-pVTZ^34^ basis set (see Section S1 for further details). To demonstrate the modularity of our approach, after optimizing the *rev*PBE-D3 parameters for the water–water interactions, we further reoptimized also the Mg^2+^-water interaction as provided by the *rev*PBE-D4 model. In this case, 40 first-hydration-shell configurations (i.e., Mg­(H_2_O)_6_^2+^) and eight second-shell configurations (i.e., Mg­(H_2_O)_6_(H_2_O)_13–17_) were extracted from classical MD trajectories and evaluated at the DLPNO–CCSD­(T)-F12 level using the cc-PVQZ basis set as implemented in ORCA.^35^ For each configuration, we computed (i) the total cluster energy and (ii) the energy of the water fragment frozen in the same geometry. The interaction energy for method X (X = DFT or DLPNO–CCSD­(T)) was obtained as follows

1 $$\Delta E_{\mathrm{int},X}=E_{\mathrm{tot},X}-E_{\mathrm{wat},X}-E_{{\mathrm{Mg}}^{2+},X}$$

Energy deviations (i.e., Δ*E*_int,DFT_ – Δ*E*_int,CC_) were minimized by tuning the *S*_8_ coefficient of the D4 potential to reproduce the metal ion–water interaction energies, while keeping the same optimized parametrization for the water–water description (see Section S1 for further details).

### Machine Learning Interatomic Potentials

We used the functionals so optimized to develop a reference database for water MLIP training using the MACE^30^ architecture (see Sections S2 and S3 for further details). The construction of the fitting database is critical in determining MLIP accuracy.^36,37^ A flowchart of the main steps of the computational protocol is illustrated in Figure 1. Initially, we generated 10 200 representative liquid-phase structures through classical MD simulations using the TIP4P/2005 ^38^ water model. To build up the training set, energies and atomic forces were evaluated at the *rev*PBE-D3^*OPT*^ level and used to train the first version of the MLIP, MLIP^(1)^. In an active learning strategy,^39^ the obtained MLIP^(1)^ model was then used to sample additional liquid water structures and enrich the original training set, from which we developed an intermediate MLIP model, MLIP^(2)^. In a second step, we used MLIP^(2)^ to generate further liquid configurations from classical MD and path-integral MD (PI-MD) simulations to ensure well-balanced performances when including nuclear quantum effects. Upon further training, the final MLIP model (i.e., the third version MLIP^(3)^ or MLIP-*rev*PBE-D3^*OPT*^) was obtained. MLIP-*rev*PBE-D3^*OPT*^ was initially validated on a large number of *rev*PBE-D3^*OPT*^ interaction energies obtained from liquid water and ice structures not included in the training set (Figure S4) and energies and forces from Born–Oppenheimer AIMD simulation of liquid water (Figure S8), achieving excellent results in terms of both interaction energies (RMSE of 0.1 meV/atom) and atomic forces (RMSE of 8 meV/Å). An analogous active learning protocol was applied to develop an effective MLIP for describing Mg^2+^ in water. A system containing MgCl_2_ in 125 water molecules was initially simulated using classical MD, then selected configurations were re-evaluated at the *rev*PBE–D^OPT^ level and used to train an initial MLIP Mg^2+^–water potential. The first MLIP model was used to carry out additional MD and well-tempered metadynamics^40,41^ simulations in which the ion–water coordination number served as a collective variable (see Section 2.5 below), efficiently exploring the water coordination space. The resulting enlarged data set was employed for the final training, yielding a MLIP that reproduced *rev*PBE–D^*OPT*^ energies and forces with the same accuracy achieved for pure water (RMSE energy per atom of 0.2 meV/atom, RMSE forces of 9 meV/Å). The same configurations (i.e., MgCl_2_ in water) were also used to develop a MLIP model based upon standard DFT-D3(0) for water–water interaction and standard DFT-D4 for Mg^2+^-water interactions for the sake of comparison, referred to as MLIP-*rev*PBE-D4. We note in passing that the parametrization procedure we exploit here could be applied not only to MLIPs, but also to classical force fields, some of which have been proven capable of reproducing the properties of water accurately.^42^

**Figure 1:**
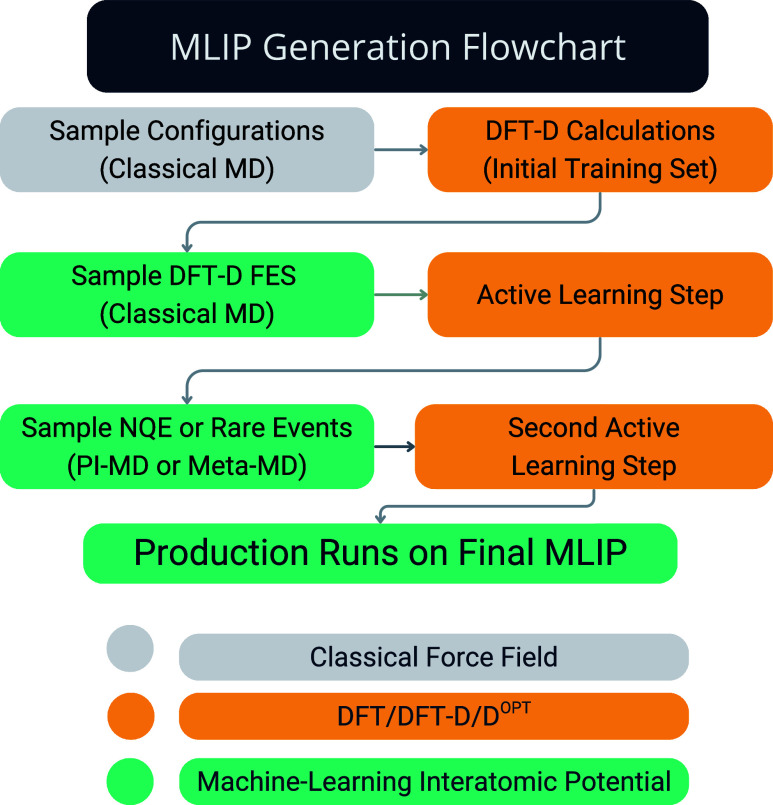
Flowchart illustrating the main steps followed during the MLIP generation. First, uncorrelated structures of liquid water or aqueous solutions were generated through classical MD simulations, evaluating interaction energy and atomic forces for each one at the *rev*PBE-D^*OPT*^ level. The initial MLIP was then used to carry out active learning in two further steps, the first one to correctly sample the free energy surface relative to the DFT model used and the second to sample nuclear quantum effect, for the case of water, or rare events (i.e., water exchange the first solvation shell) for the case of Mg^2+^ aqueous solutions.

### Molecular Dynamics Simulations

Production Molecular dynamics (MD) simulations were performed using machine learning interatomic potentials (MLIPs) based on the MACE framework.^30^ Classical simulations of bulk liquid water contained *N* = 510 molecules in a cubic box. The equations of motion were integrated with LAMMPS^43^ using a 0.5 ^44^ fs time step. The starting system was first equilibrated in the *NVT* ensemble for 1 ns using a Langevin thermostat. Structural observables—radial distribution functions (RDFs), hydrogen–bond angle distributions, and the tetrahedral order parameter *q*—were extracted from separate *NVT* runs, while dynamic quantities (diffusion coefficients, vibrational density of states, IR spectra) were evaluated from *NVE* trajectories (see SI, Section S4 for more details). Density–temperature curves (Figure 4) were obtained from *NpT* trajectories of 2.5 ns under a Langevin thermostat and barostat. Equilibrium properties were averaged over the final ns.

Quantum nuclear effects were included through either path–integral MD (PI-MD) with the PIGLET scheme^45^ or Thermosttated Ring-Polymer Molecular Dynamics^46^ (TRPMD) as implemented in i-PI^47^ and using ASE^48^ implementation of MACE to evaluate energy and forces. The systems consisted of 64 water molecules represented by 56 beads using a time step of 0.25 fs. A PIGLET generalized Langevin thermostat was applied to the centroid and internal modes to replicate *NVT* conditions, while *NpT* simulations used an additional isotropic Langevin barostat.

### Thermodynamic and Dynamical Properties

The vaporization and fusion enthalpies were evaluated from the following expressions

2 $$\Delta H_{\mathrm{fus}}=H_{l}-H_{s}=\left(\left\langle U_{l}\right\rangle -\left\langle U_{s}\right\rangle \right)+\mathrm{PV}$$

3 $$\Delta H_{\mathrm{vap}}=H_{g}-H_{l}=\left(\left\langle U_{g}\right\rangle -\left\langle U_{l}\right\rangle \right)+\mathrm{RT}$$

where ⟨*U*_*x*_⟩ denotes the ensemble-average internal energy of the system, with *s* the solid, *l* the liquid, and *g* the gas-phase. Energy averages were obtained from 200 ps *NVT* simulations, starting from initial configurations previously equilibrated under *NpT* conditions. The *PV* and *RT* terms were set from the corresponding control values of the relative *NVT* and *NpT* simulations. Isobaric heat capacity, *c*_*p*_ and thermal expansion coefficient α_*P*_ defined as

4 $$c_{p}={\left(\frac{\partial H}{\partial T}\right)}_{P}$$

5 $$\alpha_{P}=-\frac{1}{V}{\left(\frac{\partial V}{\partial T}\right)}_{P}=-\frac{1}{\rho }{\left(\frac{\partial \rho }{\partial T}\right)}_{P}$$

were evaluated following the procedures described in Reddy et al.^16^ Diffusion coefficients were calculated at 280, 300, and 320 K by linear regression of the mean square displacement (MSD) slopes, excluding the initial and final picoseconds to focus solely on the diffusive regime.

6 $$D_{L}=\lim_{t\to \infty }\frac{1}{6t}\left\langle |r\left(t\right)-r\left(0\right){|}^{2}\right\rangle$$

For classical MD simulations at 300 K, diffusion coefficients were averaged over 10 simulation replicas of 180 ps each. At 280 and 320 K, averages were obtained from 10 replicas of 10 ps each. The TRPMD simulations used five replicas of 8 ps per temperature point. Errors were estimated via bootstrap resampling, with corrections applied to account for finite-size effects of the simulation cell box.^49^

7 $$D_{\infty }=D_{L}+\frac{k_{B}T\xi }{6\pi \eta L}$$

The vibrational density of states (vDOS) was obtained from *NVE* trajectories for classical dynamics and using TRPMD to include nuclear quantum effects. We ran 13 (30 ps) independent simulations for TRPMD and 10 (75 ps) for classical MD simulations. The vDOS was computed by applying a Hann window to the velocity autocorrelation function, followed by a Fourier transform. The resulting spectra were corrected for quantum effects and further adjusted according to the procedure described in Melani et al.^50^

8 $$\mathrm{vDOS}\left(\omega \right)=\left(\frac{\hbar \omega }{k_{B}T}\cdot \frac{1}{1-e^{-\hbar \omega /k_{B}T}}\right)\cdot \int_{0}^{T_{\max}}C_{\mathrm{vv}}\left(t\right)\cdot \sin^{2}\left(\frac{\pi t}{T_{\max}}\right)\cdot \cos\left(\omega t\right)dt$$

where *T*_max_ is the total length of the time window and *N* is the total number of atoms in the system. *C*_*vv*_ is the velocity–velocity autocorrelation function

9 $$C_{\mathrm{vv}}\left(t\right)=\frac{1}{N}\sum_{i=1}^{N}\left\langle v_{i}\left(0\right)\cdot v_{i}\left(t\right)\right\rangle$$

Finally, the spectra were averaged for different runs.

### Free Energy of Ion Coordination

To evaluate the free energy change associated with the first–shell water coordination, we adopted the water coordination number *s*, as described in refs 51,52 , as a continuous collective variable defined as follows

10 $$s=\sum_{i=1}^{N}\left[1-\frac{1}{1+\exp\left[a\left(r_{i}-r_{0}\right)\right]}\right]$$

where *r*_*i*_ is the ion–oxygen distance of the *i*-th solvent molecule, *N* is the total number of water molecules in the simulation box, *r*_0_ denotes the ion–oxygen cutoff distance, and *a* (=4.0 Å^–1^) controls the steepness of the Fermi–type switching function. The cutoff *r*_0_ was set on the basis of the first minimum of the ion–oxygen radial distribution function (in this case, *r*_0_ = 3.0 Å). An extensive sampling with well-tempered metadynamics^40,41^ (computational details of the meta-MD simulations can be found in the Supporting Information in Section S4) was carried out using LAMMPS in combination with the PLUMED library (v2.9.0).^53,54^

## Results and Discussion

### DFT-D Optimization

In the present work, we adopted the *rev*PBE GGA exchange-correlation functional^55^ and performed a careful reoptimization of the D3(0)^56^ potential since the standard *rev*PBE-D3 reported already quite satisfactory results on various properties of water under different physical conditions,^57–60^ although the good performance of *rev*PBE-D3 for liquid water could be due to a favorable compensation between functional-driven and density-driven errors.^61^ Note that *rev*PBE-D3 resulted the best performing DFT approximation toward the DMC-ICE13 data set.^11^ Besides, *rev*PBE-D3 is better suited for AIMD simulations or large-scale calculations than recently proposed hybrid or meta-GGA functionals, due to its computational efficiency. Dispersion corrections were optimized against DMC benchmark data.^12^ A MACE MLIP was then carefully trained against *rev*PBE data via a judicious choice of the training database (see Section 2.2 and Figure 1), and then assessed for its ability to predict key structural, thermodynamic, and dynamic properties of water, ice, and aqueous solutions of Mg^2+^, providing a remarkable agreement with available experimental data, as discussed in the following.

The quality of the refined *rev*PBE-D3 model (hereafter referred to as *rev*PBE-D3^*OPT*^) is illustrated in Figure 2 where relative errors in the interaction energy per molecule (MAE/mol) were compared to the results issued from the standard *rev*PBE, *rev*PBE-D3/D4 ^62^ and the hybrid *rev*PBE0-D4 on various systems, including: (i) water clusters of growing size (i.e., 9-mer, 15-mer, and 27-mer),^64^ (ii) liquid water configurations (used in the optimization step),^12^ and (iii) different ice polymorph structures from the ICE13 data set.^11^ Our optimized model consistently improved the estimated interaction energy across all examined systems, from small water clusters to multiple liquid and ice structures, reaching a remarkably low MAE/mol of less than 0.1 kcal/mol for the liquid phase (i.e., within DMC intrinsic statistical error). Note that energy deviations remained significantly small throughout the individual structures of the benchmark data sets, showing a noticeable improvement compared to the standard D3 and D4 parametrization (Figures S3–S5). Similar results were obtained on test calculations on the optimized neutral water clusters of the WATER27 data set^65^ (MAE/mol: 0.11 kcal/mol, while D3 and D4 reported 0.21 and 0.20 kcal/mol, respectively). Besides, the present optimized model provided the same very good agreement as the standard *rev*PBE-D3 functional with the recent DMC-ICE13 ice polymorph energies,^11^ showing an error of only 0.19 kcal/mol per molecule. The optimization of the magnesium-water interaction is also reported in Figure 2 showing a significant increase in accuracy with respect to the default values. We have optimized the empirical D4 correction for the interaction between Mg^2+^ and water changing the S_8_ parameter to minimize the error, with respect to the reference data, in the interaction energy reported in eq 1. This was done using only two-shell structures as a reference because they are more representative of the environment observed in molecular dynamics. While the standard parametrizations of the D corrections have proven to be sufficiently accurate for water–water interactions, this is not true for the Mg^2+^-water interaction. In Figure 2, we show how standard parametrizations do not mitigate the initial error of the DFT (MAE/mol­(DFT): 2.05 kcal/mol MAE/mol­(DFT-D4): 1.42 kcal/mol). To obtain good agreement (MAE/mol­(DFT-D^*OPT*^): 0.09 kcal/mol) with the DLPNO–CCSD­(T)-F12 reference calculations, we increased the value of the S_8_ parameter from 1.75 to 9.01. In Figure S5 we report the deviations with respect to DLPNO–CCSD­(T)-F12 for all the single and double shell cluster structures. The MAE on the single-shell Mg­(H_2_O)_6_^2+^ clusters rises to 1.21 kcal/mol, indicating a slight overbinding caused by tuning on the larger set, yet the optimized functional still outperforms plain *rev*PBE (MAE/mol: 2.98 kcal/mol) and the default D4 variant (MAE/mol: 1.79 kcal/mol) by a comfortable margin. A recent study^66^ demonstrates that DC-SCAN is capable of representing ion–water clusters with near-chemical accuracy, albeit at significantly higher computational cost (i.e., meta-GGA on HF densities). In this work, the ad hoc optimization of D4 yielded a system-specific *rev*PBE-D4 model that attains negligible errors in the ion–solvent interaction energy, comparable to those reported by Palos et al.,^66^ at lower cost, thus enabling longer time scales and larger systems. Accordingly, our protocol is advantageous when high-throughput or large-scale simulations are needed, where the one-time optimization cost is amortized.

**Figure 2:**
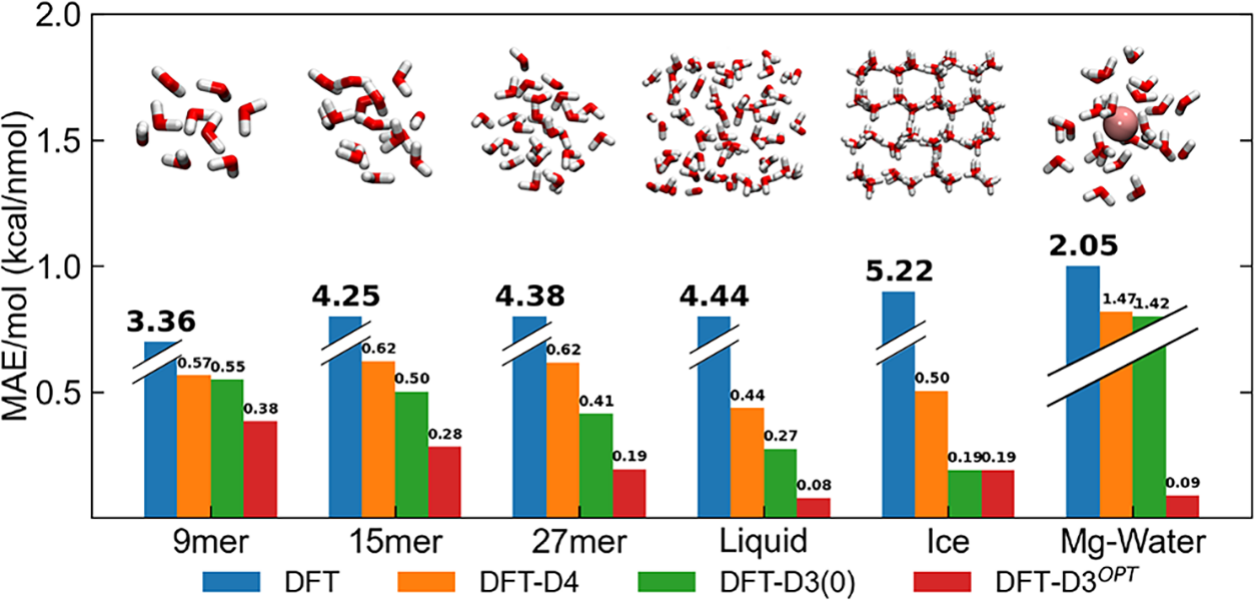
Mean absolute error (MAE) per molecule of the interaction energy computed using the standard *rev*PBE-D4, *rev*PBE-D3, and reoptimized *rev*PBE-D3^*OPT*^ (or D4 for the Mg-Water interaction) model with respect to DMC on isolated water clusters of growing size (i.e., 9-mer, 15-mer, and 27-mer),^64^ liquid-phase structures (including 32 and 64 water molecules),^12^ ice polymorph structures from the DMC-ICE13 ^11^ data set. The references for the Mg-Water systems were evaluated at the DLPNO–CCSD­(T)-F12 with the cc-PVQZ basis set

### MLIP Simulations of Bulk Water and Ice

The MLIP-*rev*PBE-D3^*OPT*^ model was then used to carry out liquid water and ice simulations under different conditions, performing both classical and quantum MD simulations. A system containing 510 water molecules was set up to reproduce the liquid-phase properties, while different systems were generated to represent the ice polymorph structures (see Section S4 for further details). As depicted in Figure 3, the computed O–O, O–H, and H–H RDFs of liquid water at normal conditions were all very close to the experimental counterparts.^67,68^ The obtained RDFs also matched very closely previous first-principle MD simulations based on the standard *rev*PBE-D3 functional,^58,59^ thus indicating that local structural properties were not much affected by the present D3 reoptimization. Moreover, the introduction of nuclear quantum effects was more noticeable in the first peak of the O–O RDF, which showed a slightly enhanced height and a better agreement with experiments, in line with the observation reported in ref 59. Figure 3d depicts the H-bond angle distribution, showing again a good agreement between simulation and experiments. In particular, at room temperature classical and PI MD simulations showed a distribution peaked at 15.0 and 13.9°, respectively (exp., 12.1°), while at higher temperature (*T* = 323 K) both distributions shifted slightly toward larger angles.

**Figure 3:**
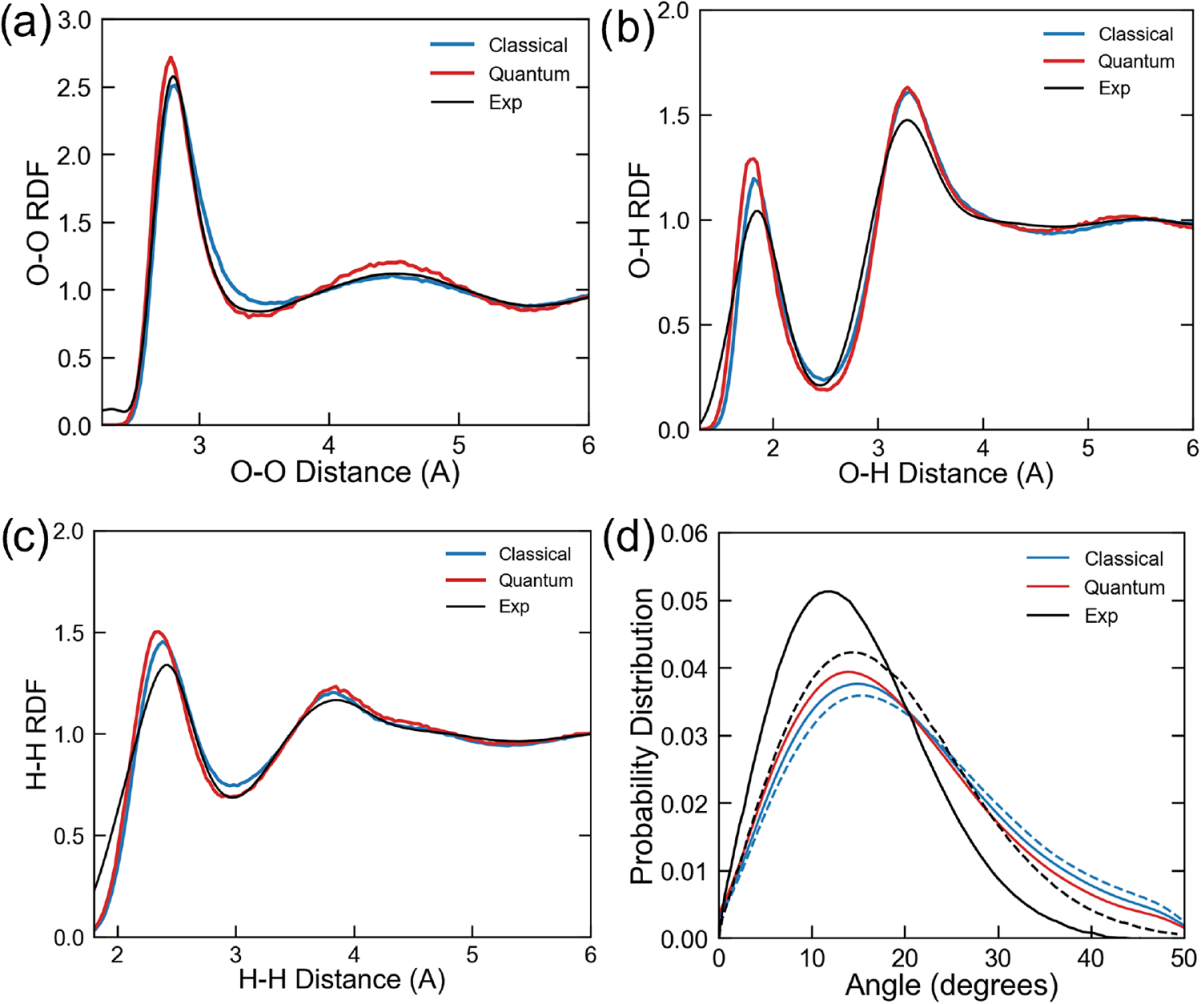
Experimental and simulated (a) O–O, (b) O–H, and (c) H–H RDFs obtained from classical and path-integral MD simulations using the MLIP-*rev*PBE-D3^*OPT*^ model. O–O Experiment was replotted from Skinner et al.,^67^ O–H and H–H were replotted from Soper et al.^68^ (d) Experimental and simulated H-bond angle distributions obtained at room temperature (solid line) and *T* = 323 K (dashed line). Experiments were extracted and replotted/reproduced from Modig et al.^69^

What is most notable, *NpT* simulations exploiting the MLIP-*rev*PBE-D3^*OPT*^ model reported an accurate density–temperature profile of both liquid water and ice. In Figure 4a, the density prediction of liquid water obtained from classical MD in the temperature range 250–330 K closely followed the experimental density (Δρ < 0.015 g/cm^3^ between 275 and 330 K). Besides, the computed density correctly displayed a maximum at 277 K (sim. 1.015 g/cm^3^, exp. 1.0 g/cm^3^), while at room temperature (300 K) the estimated density was 1.008 g/cm^3^ (exp. 0.997 g/cm^3^, Δρ = 0.01 g/cm^3^). Below 273 K, in the supercooled water regime, we noticed somewhat larger discrepancies from experiments, though they remained rather limited (Δρ < 0.02 g/cm^3^). The PI-MD simulations predicted a slightly larger density than classical MD over the same temperature range (275–330 K), although the difference is not significant after taking into consideration the increased statistical error (σ = 0.03 g/cm^3^), hence again satisfactorily close to the experimental densities. It is worth noting that the reoptimized *rev*PBE-D3^*OPT*^ model outperformed previous first-principle and MLIP simulation studies based on various DFT functionals.^57^ For comparison, two of the most recent water simulation studies, one based on a neural network trained on standard *rev*PBE0-D3 functional^7^ and another one on a many-body potential derived from the density-corrected SCAN model,^13^ underestimated the density–temperature dependence (Figure S9), while showing an overall reliable prediction for liquid and solid water. Similarly, the AIMD simulation by Pestana et al., employing the *rev*PBE-D3 approximation, provided a density of about 0.97 g/cm^3^ at ambient conditions.^58^ Indeed, simulations carried out in this work using a MLIP trained on the unmodified *rev*PBE-D3 functional predict a density profile versus temperature well in line with the ab initio results of ref 58 (Figure 4a), confirming that the original D3 is somewhat underbinding. On the contrary, our reoptimized model showed a nearly perfect match with the density isobars (and the related thermal expansion coefficient, see Figure 5d below) issued from the MB-pol^16,18^ many-body potential (Figure S9), which represents a gold standard for modeling physical properties of water. It is worth noting that the slight increase in liquid density issuing from our model with respect to standard *rev*PBE-D3 reflects well the improved water interaction energy of the former, as shown in Figure 4a. Furthermore, in a previous work, Montero De Hijes et al.^57^ showed how the density isobar and the melting temperature of water are highly sensitive to the damping function of the D3 term. Their heuristic revPBE0–D3/mix model (i.e., a mix of the zero-damping and Becke-Johnson damping functions) reproduced these properties well, but it was flagged as potentially nontransferable.

**Figure 4:**
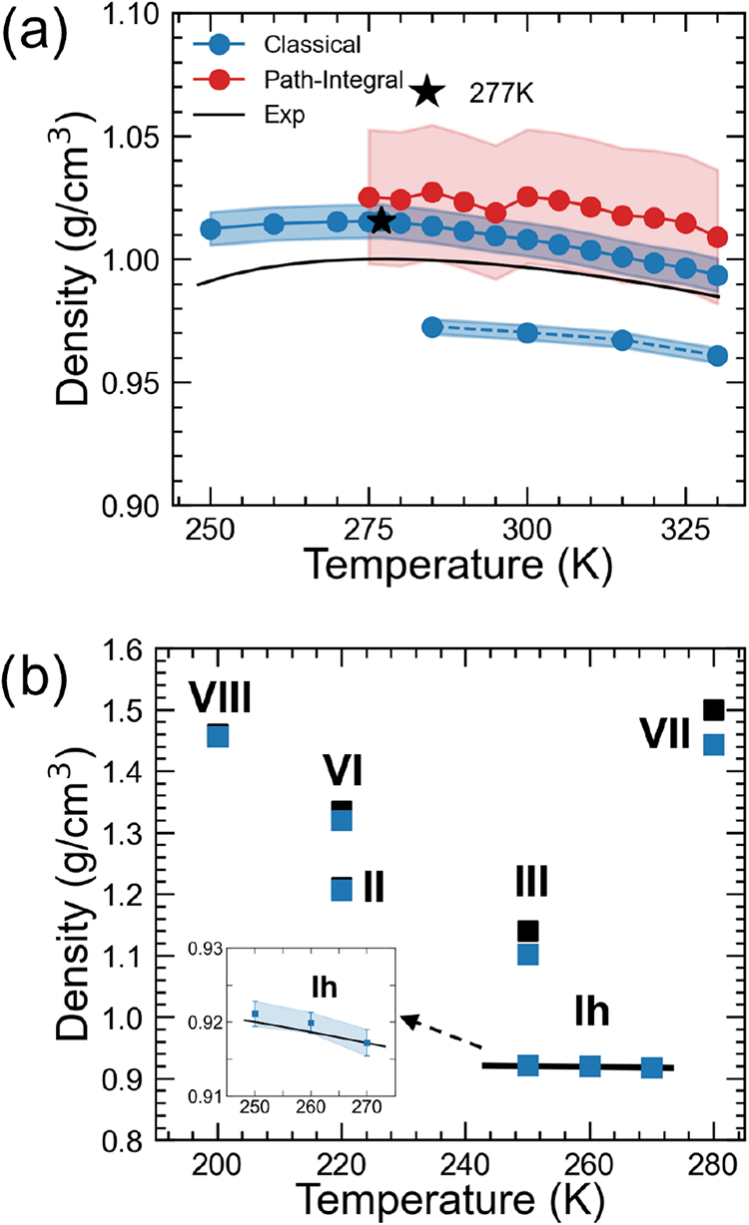
(a) Experimental and simulated density isobars of liquid water evaluated from classical and path-integral MD simulations using the MLIP-*rev*PBE-D3^*OPT*^ model. A star indicates the density maximum from classical MD simulations. Results from the unmodified *rev*PBE-D3 model are also reported (dashed line). Experimental densities were replotted from NIST.^70^ See also Figure S9 of the Supporting Infromation for a comparison with other simulations. (b): Experimental and simulated (classical MD) densities for several ice polymorphic phases evaluated at ambient pressure and stability temperature. Inset, zoomed view of the Ih ice phase density at different temperatures. Experimental densities of ice III, VII, and VIII were replotted from ref 71, ice II and ice VI from ref 72, and ice Ih from NIST.^70^

**Figure 5:**
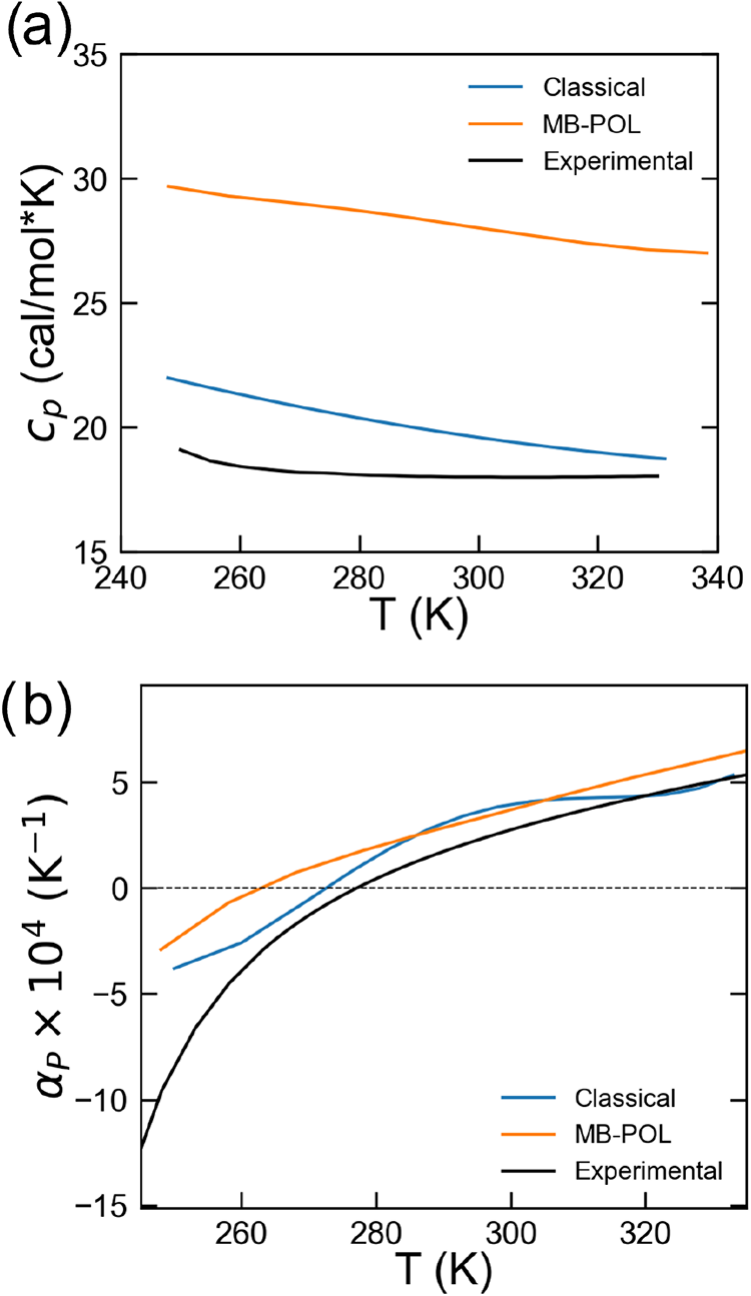
(a): Experimental and simulated isobaric specific heat capacity from classical MLIP-*rev*PBE-D3^*OPT*^ and MB-Pol extracted and replotted from.^16^ Experimental values were replotted from Kell.^73^ (b): Experimental and simulated thermal expansion coefficient isobars from classical MLIP-*rev*PBE-D3^*OPT*^ and MB-Pol extracted and replotted from.^16^ Experimental values were replotted from Kell.^73^

Furthermore, we carried out *NpT* simulations on six different ice phases (i.e., Ih, II, III, VI, VII, and VIII) at ambient pressure and relative stability temperature. As shown in Figure 4b, the predicted densities were again in excellent agreement with available experimental data. In particular, the ice phase Ih, II, VI, and VIII exhibited negligible density deviations (≤0.01 g/cm^3^), while III and VII reported larger but limited discrepancies, below 0.06 g/cm^3^. Moreover, MLIP simulations also captured minor physical changes, such as the slight density reduction in the temperature range 250–270 K of ice phase Ih (inset of Figure 4b).

In addition to the above structural properties, the MLIP-*rev*PBE-D3^*OPT*^ model ensured very satisfactory results when tested on thermodynamic quantities, namely the enthalpy of melting (Δ*H* ^fus^) and vaporization (Δ*H* ^vap^) obtained according to eqs 2 and 3. The predicted Δ*H* ^fus^ was 1.67 kcal/mol (exp. 1.44 kcal/mol), and Δ*H* ^vap^ was 10.63 kcal/mol (exp. 10.49 kcal/mol), in remarkable agreement with experiments.

The specific heat at constant pressure, *c*_*p*_, and the isobaric thermal-expansion coefficient, α_*P*_, were also extracted from our *NPT* simulations according to eqs 4 and 5. Figure 5a shows *c*_*p*_(*T*) from the MLIP-*rev*PBE-D3^*OPT*^ and the many-body MB-Pol^16^ model alongside experiments. Our model somewhat overestimated *c*_*p*_ at low temperature (<290 K) while approaching experiments at room temperature and beyond. This excess heat capacity at low *T* likely reflected anharmonic motions that are too facile in the absence of nuclear quantum effects. Noteworthy, our model outperformed MB-Pol in the same investigated temperature range (Figure 5a). Moreover, Figure 5b reports the isobaric thermal-expansion coefficient α_*P*_, plotted as α_*P*_ × 10^4^ K^–1^ for convenience. The MLIP-*rev*PBE-D3^*OPT*^ reproduced well the anomalous negative thermal expansion below the density maximum, while crossing zero at 277 K. These results confirmed the accurate description of the water density in a rather extended temperature range, as compared to experiments.

Finally, we performed *NVE* and TRPMD simulations from which we evaluated dynamical properties such as the self-diffusion coefficient and the vibrational density of states (vDOS) of liquid water. In Figure 6a, the self-diffusion coefficient, as obtained according to eq 6 and corrected for infinite size according to eq 7 of water structures using both classical and path-integral MD, is depicted as a function of temperature. Results show how the inclusion of quantum effects significantly improved the description of water mobility. The diffusion coefficient was overestimated at the classical level (at 300 K, 3.05·10^–9^ m^2^ s^–1^) but well-reproduced at the path-integral MD level (2.27·10^–9^ m^2^ s^–1^, exp: 2.41·10^–9^ m^2^ s^–1^). In Figure 6b, we compare the vDOS obtained according to eqs 8 and 9 with the experimental IR power spectrum of water. The vDOS was evaluated according to the procedure described in.^50^ In classical MD, the O–H stretching frequency was slightly blue-shifted relative to experimental data, whereas PI-MD resulted in a red-shift, showing a discrepancy of ≈1.5% on peak position. These trends were consistent with previous AIMD studies employing the *rev*PBE-D3 functional.^58,59^ In particular, quantum nuclear effects red-shifted the O–H stretching peak position by about 250 cm^–1^, similar to the AIMD results obtained in ref 59. Concerning the observed deviation from experiments, this is likely due to a still excessive “softness” of the O–H bond in *rev*PBE-D3^*OPT*^ with respect to the exact or hybrid-functional.

**Figure 6:**
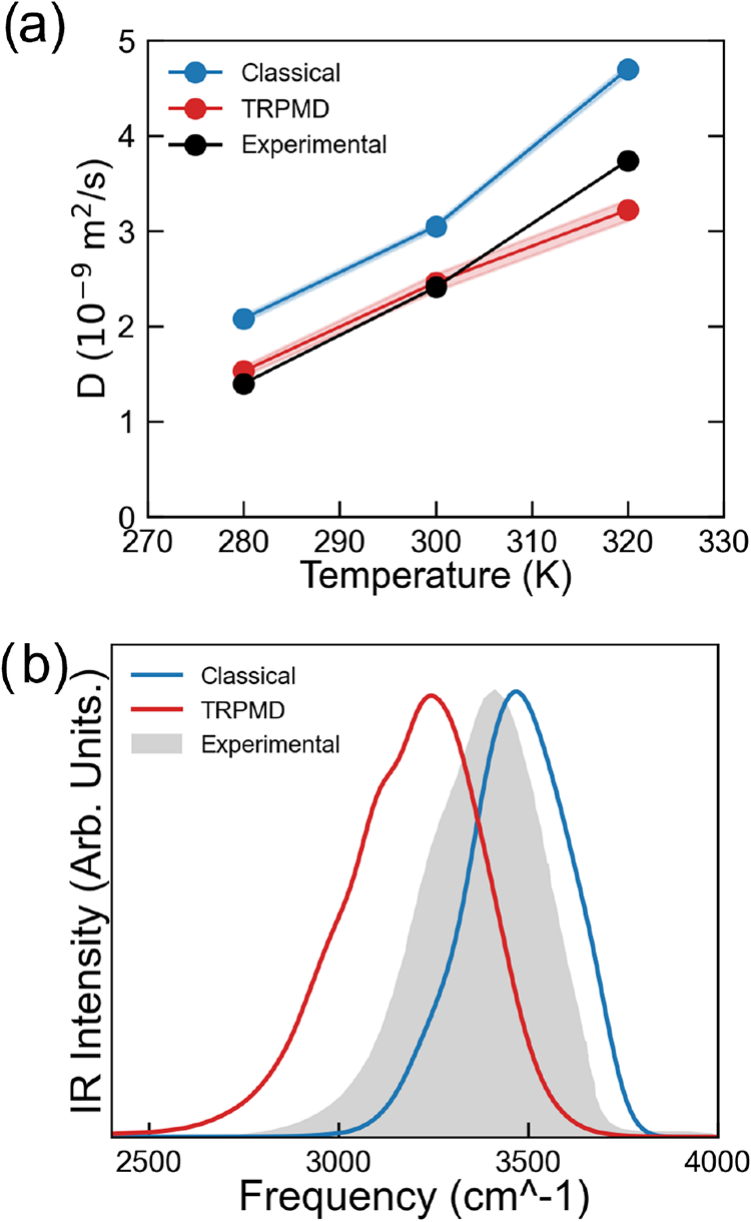
(a): Experimental and simulated Diffusion Coefficient isobars for water from classical and PI-MD simulation using MLIP-*rev*PBE-D3^*OPT*^. Experimental data were replotted from Prof. Dietrich’s Web site https://dtrx.de/od/diff/. (b): Experimental IR Spectrum and simulated vDOS rescaled to experimental density for comparison from classical and PI MD simulation employing MLIP-*rev*PBE-D3^*OPT*^. Experimental values were extracted and replotted from Bertie et al.^74^

### Mg^2+^ in Aqueous Solution

The MLIP-*rev*PBE-D^*OPT*^ model was further tested to perform MD simulations of Mg^2+^ in water. In Figure 7a, the Mg–O RDF obtained from our MLIP simulation is depicted along with results issuing from other popular classical force fields, namely the MicroMg^75^ and the 12–6–4 from Li and Merz,^76^ as well as a MLIP derived from standard dispersion corrections (i.e., MLIP-*rev*PBE-D). The MLIP-*rev*PBE-D^*OPT*^ predicted well the position of the first RDF peak at 2.07 Å, matching experiments within the statistical error (2.09 ± 0.04 Å).^77^ Note that our MLIP, similarly to other recent ML models,^78,79^ showed a broader first peak in comparison to classical force fields, thus indicating a more flexible first solvation shell. The beneficial effect of the optimization first became apparent in the description of the second solvation shell, since MLIP-*rev*PBE-D^*OPT*^ reported the second Mg–O peak appreciably closer to experiments than both the unoptimized DFT-D4 model and the classical force fields. The optimization also improved the predicted density with respect to the other tested models, as reported in Table 1. Upon reoptimization, the obtained MLIP-*rev*PBE-D^*OPT*^ displayed a density of 0.99 ± 0.01 g/cm^3^, in close agreement with experiments (1.005 g/cm^3^ from Al Ghafri et al.^80^), while the same property was somewhat underestimated by the default parametrization. In a previous work, Kostal et al.^81^ observed that, in the case of monovalent cations, the use of D3 can worsen the description of structural properties due to overbinding of the pristine *rev*PBE functional. Here, we showed how, in the case of Mg^2+^, the use of a reliable DFT approach in combination with Dx reoptimization allowed us to recover the correct structural, thermodynamic, and kinetic behavior.

**Table 1:** Experimental and Simulated Exchange Kinetics in the First Solvation Shell and Aqueous Solution Density

model	ρ [g cm^–3^]^a^	Δ*A* ^‡^ [kJ mol^–1^]	*k*_*X*_ [s^–1^]
MicroMg (ref)	0.99	34.9	8.0 × 10^5^
*rev*PBE–D4	0.95	23.3	8.4 × 10^7^
*rev*PBE–D^OPT^	0.99	32.6	2.0 × 10^6^
Exp	1.005^b^		6.7 × 10^5^ ^c^

a Density of a 0.109 M aqueous solution of MgCl_2_.

b Experimental density was taken from Al Ghafri et al.^80^

c Experimental rate was taken from Bleuzen et al.^82^

**Figure 7:**
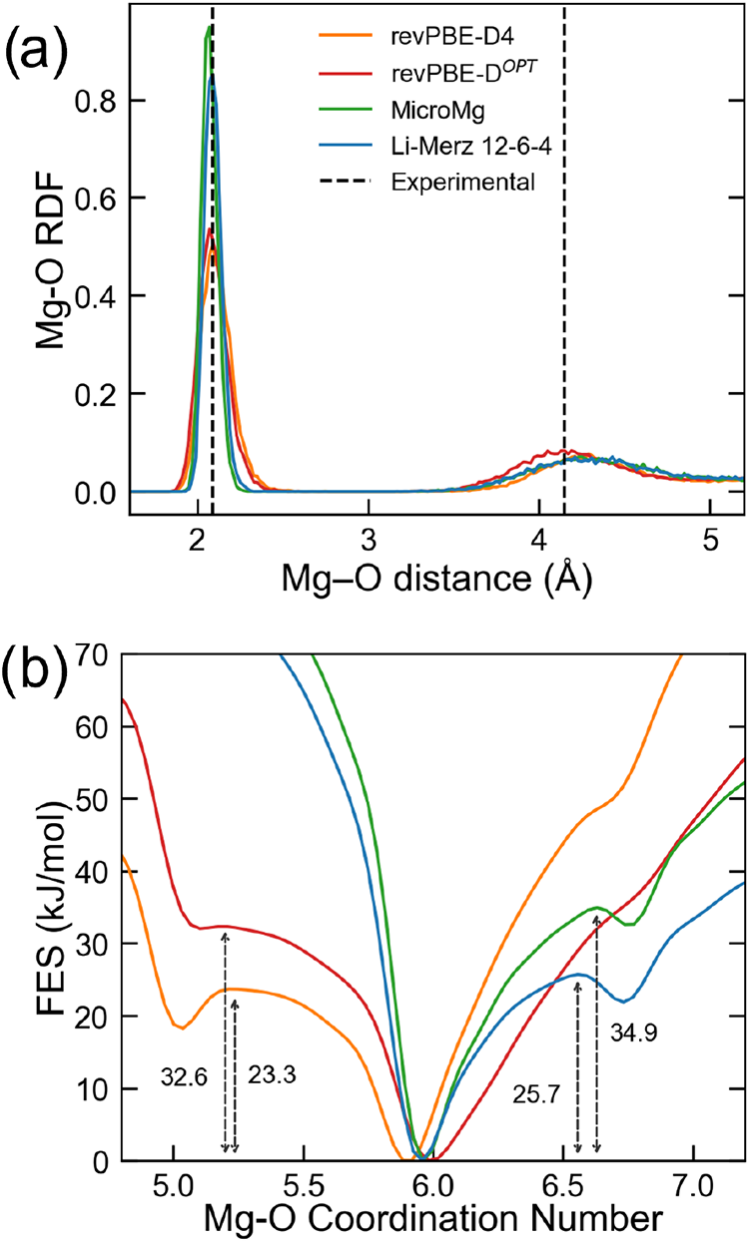
(a): Experimental and simulated Mg–O RDF as obtained from MLIP-*rev*PBE-D4, D^*OPT*^ and from MicroMg^75^ and Li-Merz^76^ 12–6–4 Classical Force Fields. Experimental reference (black dashed line 2.09 ± 0.4), was replotted from Marcus.^77^ (b): Free Energy Surface of Mg^2+^-Water coordination as obtained from MLIP-*rev*PBE-D4, D^*OPT*^ and from MicroMg and Li-Merz 12–6–4 Classical Force Fields.

Switching to dynamic properties, we focused on the free-energy barrier for water exchange in the first solvation shell. To this purpose, we evaluated the free energy change as a function of the ion–water coordination number (as defined in eq 10) as obtained from purposely performed meta-MD simulations according to the computational protocol described in refs 51,52 (see Free Energy of Ion Coordination section for further details), and we report the results of these simulations in Figure 7b, where we compare DFT and force-field predictions for this quantity. As apparent from Figure 7b, all meta-MD simulations predicted the 6-fold water coordination as the most stable for Mg^2+^. However, importantly, only the DFT-based potentials correctly indicated a dissociative water exchange mechanism with a 5-fold water configuration as the second most favorable state (a shallow local minimum in the free-energy). A dissociative water exchange mechanism is indeed consistent with the findings reported in the NMR experimental study by Bleuzen et al.^82^ In contrast, both classical force-field simulations predicted an erroneous associative mechanism through a hepta-coordinated state, possibly due to the lack of many-body interactions. It is also worthwhile to observe that, only using a MLIP parametrized against a DFT functional in which the dispersion terms have been reoptimized, i.e., only using MLIP-*rev*PBE-D^*OPT*^, the simulations were able to reproduce fairly well also the predicted energy barrier for water exchange and the experimental rate constant for the process (Table 1). Here, we assume as the reference energy barrier the one obtained from the MicroMg^75^ parametrization (34.9 kJ/mol), a model purposely developed to match the experimental water exchange rate. The rate constants were estimated by considering an exponential scaling factor with respect to the MicroMg model (*k*_X/kMicroMg_ = exp­(ΔΔ*A* ^‡^/*kT*)). Noteworthy, the default *rev*PBE–D4 model underestimated the barrier by about 12 kJ/mol, hence overshooting the predicted rate constant by 2 orders of magnitude (Figure 7b and Table 1). We thus conclude that a physically sound re–parametrization of the D4 term is necessary and sufficient to restore a realistic description of the first–shell solvation structure around the magnesium ion and its water exchange kinetics, simultaneously reproducing the experimentally established dissociative mechanism. Note that our protocol is not only computationally effective but also easily transferable to other mixture and/or aqueous solutions in comparison with state-of-the-art gold standard approaches, e.g., MB-pol.

## Conclusions

In summary, the key features of our proposed in silico strategy are (1) fine-tuning a computationally efficient DFT-D (GGA) functional enhanced by dispersion corrections (MAE/mol <0.1 kcal/mol) toward high-fidelity reference data; (2) building a proper training database to generate an effective MLIP able to reproduce interaction energies and atomic forces of the optimized DFT-D model with great accuracy (RMSE of 0.1 meV/atom and 8 meV/Å); (3) validating the predictive capability of the trained MLIP over a wide ensemble of physicochemical properties of diverse systems, from nanoclusters to bulk liquid and solid phases.

In particular, our results demonstrate, first, that system-specific optimization of dispersion parameters can be successfully extended to treat neat water and aqueous solutions. By then incorporating the resulting high-quality energy and gradient evaluations into a data-driven neural network potential, the MLIP-*rev*PBE-D3^*OPT*^ model, accurate MD simulations of complex condensed-phase systems can be carried out at an affordable computational cost. Notably, the so obtained MLIP-*rev*PBE-D3^*OPT*^ model is able to reproduce the energetics of the dispersion-corrected DFT approach for a diverse ensemble of systems, from small clusters to bulk liquid and solid phases. It also successfully predicted a wide range of experimental equilibrium and dynamic quantities such as radial distribution functions, self-diffusion coefficients, vibrational density of states, enthalpy of melting and enthalpy of vaporization, and, impressively, the mass density of water in several phases, ranging from the liquid phase (matching the observed maximum at 277 K) to various forms of ice as evaluated at different temperatures. The protocol extends naturally to interfacial systems (e.g., solid–liquid, liquid–vapor) by harvesting snapshots from coexistence or slab simulations at the target *T*-*P* conditions and inserting them (via the same active-learning cycle used for PIMD and Mg^2+^ metadynamics) into the fitting database.

Finally, in view of applications to, e.g., saline aqueous solutions that do require an independent optimization of solvent–solvent and solute–solvent interactions, we have tested the case of Mg^2+^ in water. Transfering the accurate description of water–water interactions into a purposedly derived MLIP for the accurate description of the metal ion–water interaction, as provided by the present approach, led to a reliable representation of both the Mg^2+^ microsolvation structure and its water exchange dynamics, which were otherwise misrepresented by classical force fields and standard (unoptimized) DFT-D models. We believe that these findings have proved the fidelity, transferability and modularity of the obtained MLIP models, thus supporting the use of the present approach for developing realistic MLIPs for a large variety of molecular systems.

## Supplementary Material



## Data Availability

All input files, trained models, and analysis scripts needed to reproduce the simulations and results are openly available at https://github.com/SNS-Brancato-Lab/Water_MG_MLIP.

## Notes

Additional details on the DFT setup, MLIP validation, performance and database sampling, and the protocols used for MD and metadynamics (Meta-MD) simulations (PDF)
