# Supplementary material for: Accurate Simulations of Water and Aqueous Solutions through Fine-Tuned Dispersion-Corrected Density Functional Theory and Machine-Learning Interatomic Potentials
Source: J Chem Inf Model. 2025 Nov 12;65(22):12437–47. doi: 10.1021/acs.jcim.5c02079 (PMC12648658; doi:10.1021/acs.jcim.5c02079)
Supplement: Supplementary file 1 [file ci5c02079_si_001.pdf]

Supporting Information for: Accurate Simulations of Water and Aqueous Solutions through Fine-Tuned Dispersion-Corrected Density Functional Theory and Machine-Learning Interatomic Potentials

Alfonso Ferretti,[†,‡] Giacomo Melani,[¶] Luca Benedetti,[†,‡] Robert A. Sorodoc,[§] Alessando Fortunelli,[*,¶] and Giuseppe Brancato[*,‖,‡]

†*Scuola Normale Superiore, Piazza dei Cavalieri 7, I-56127 Pisa, Italy*

‡*Istituto Nazionale di Fisica Nucleare (INFN), Sezione di Pisa, Largo Pontecorvo 3, I-56127 Pisa, Italy*

¶*Consiglio Nazionale delle Ricerche, CNR-ICCOM, Pisa 56124, Italy*

§*NEST Istituto Nanoscienze-CNR and Scuola Normale Superiore, 56127 Pisa, Italy*

‖*Scuola Normale Superiore and CSGI, Piazza dei Cavalieri 7, I-56127 Pisa, Italy*

E-mail: alessandro.fortunelli@cnr.it; giuseppe.brancato@sns.it

Contents

S1 DFT-D Optimization **S3**

S2 Machine Learning Interatomic Potentials **S5**

S3 MLIP Database Sampling **S6**

S4 MD Simulation Details **S7**

S5 Metadynamics **S9**

List of Figures

S1 **Figure S1:** Comparison of Interaction Energy from Plane-Wave DFT (60 vs 80 Ryd Cutoff) and Atom-Centered Gaussian Orbitals S10

S2 **Figure S2:** Effect of Different Grimme's Dispersion Models on Liquid Water Structure S11

S3 **Figure S3:** Interaction Energy Deviations with Respect to DMC: Cluster Structures S12

S4 **Figure S4:** Interaction Energy Deviations with Respect to DMC: Periodic Structures S13

S5 **Figure S5:** Interaction Energy Deviations with Respect to DLPNO-CCSD(T)-F12: Mg^{2+}-water clusters S14

S6 **Figure S6:** Grimme's Empirical Dispersion Correction of a Water Dimer . . S15

S7 **Figure S7:** RMSE of Energy and Forces versus Epochs in MLIP Training . S16

S8 **Figure S8:** Energies and Atomic Forces Comparison between MLIP and DFT S17

S9 **Figure S9:** Liquid Water Density: Comparison of MLIP-*rev*PBE-D3^{OPT} with Previous Studies and Experiments S18

S10 **Figure S10:** O-O RDF up to 1 nm distance S19

S1 DFT-D Optimization

DFT Electronic structure calculations were performed using the plane-wave basis set implementation in Quantum Espresso.[1] We adopted the revPBE[2] exchange-correlation functional with ultrasoft pseudopotentials,[3] employing energy cutoffs of 60 Ry for the plane-wave basis and 300 Ry for the electron density. To ensure convergence, a set of structures of a 9-mer water cluster was tested up to 80 Ry (wavefunctions) and 400 Ry (density). From Figure S1, we obtained a mean absolute error (MAE) of the order of 10^{-3} kcal/mol per molecule between simulations with 60 and 80 Ryd cutoff, thus showing basis set convergence. For the evaluation of the one-body term, individual water molecules were placed in a cubic box of 10 Å edge, while for 9-mer cluster structures a box of 20 Å of side length was used. Figure S1 also reports the comparison of energies between plane-wave and atom-centered Gaussian orbital calculations, employing the aug-cc-PVTZ[4] basis set. The energy deviation (MAE) between the two methods was less than 0.1 kcal/mol per molecule. Dispersion corrections were applied to the Kohn-Sham DFT energy using the Python implementation of the D3[5] and D4[6–8] methods. The reference data set of the Mg-Water clusters was generated in two stages. Four different equilibrium trajectory of bulk liquid water containing a single Mg^{2+} ion was produced with classical molecular dynamics (MD) at 298 K and 1 atm using different force fields. From that trajectories we extracted two categories of microsolvated clusters that were later used for quantum-chemical single-point calculations. We extracted 40 single-shell structures with 6 water molecules and ion and 8 double-shell structures with a number of water molecules ranging from 19 to 23. The structures were then evaluated at the DLPNO-CCSD(T)-F12 level as implemented in ORCA,[9] and all atoms were described using the cc-PVQZ dunning basis set. In this study, we specifically adopted the D3 dispersion correction with the "zero" damping function (D3(0)) for water-water intermolecular interactions, since the D4 method,[8] owing to the Becke-Johnson (BJ) damping function[10] used in the latter, led to significant over-structuring, as also noticed in refs.[11,12] In Figure S2, we report the radial distribution functions (RDFs) and the tetrahedral structure factor (q) ob-

tained from classical MD simulations using machine learning interatomic potentials (MLIPs) trained at the revPBE level, with the addition of dispersion corrections D3(0), D3(BJ), and D4 on-the-fly. The revPBE-D3^{OPT} RDF data were generated using a MLIP trained on the same model. Results show how the BJ damping function, common to both D3(BJ) and D4, led to liquid water over-structuring. In contrast, the D3(0) damping function compensated this tendency, leading to a remarkable agreement with experimental data. On the other hand, the nearly complete overlap between the RDFs generated from revPBE^{OPT} and revPBE-D3(0) simulations confirmed that the D3 reoptimization had no significant effect on local structural properties. Note also that the standard revPBE-D3(0) provided better water cluster energies compared to revPBE-D4(BJ), as shown in Figure 2 of the main article.

The optimization procedure of water-water interactions relied on diffusion Monte Carlo (DMC) reference data comprising 75 periodic liquid water structures containing 32 molecules and 18 structures containing 64 molecules from Alfè et al.[13] Following the computational protocol originally proposed in refs.,[14,15] we increased the S8 parameter from 1.01 to 1.21 to minimize the error with respect to DMC. We also tested how the model performs on water cluster structures of growing size (i.e., 9mer, 15mer, and 27mer clusters) from Alfè et al.[16] and on ice structures taken from the DMC-ICE13[17] dataset, which includes 13 different polymorphic phases. An extended analysis complementing Figure 1 is provided in Figures S3 and S4, illustrating energy deviations relative to benchmark DMC data across different systems, going from small clusters to bulk liquid and ice. Figure S3 also includes results obtained using revPBE0[18]-D4 with the aug-cc-pVTZ basis set, previously reported in ref.[15] Specifically, revPBE0-D4 exhibits mean absolute errors per molecule (MAE/mol) of 0.35 kcal/mol for 9-mer clusters, 0.37 kcal/mol for 15-mer clusters, and 0.36 kcal/mol for 27-mer clusters. While revPBE-D3^{OPT} performs similarly to revPBE0-D4 on 9-mer clusters (0.38 kcal/mol), it significantly outperforms revPBE0-D4 for larger clusters, achieving an MAE of 0.28 kcal/mol for 15-mer structures and 0.19 kcal/mol for 27-mer structures. This is in line with the goal of tailoring the optimization specifically toward condensed-phase

systems, where revPBE-D3^{OPT} performed exceptionally well (Figure S4). The MAEs of all calculations reported in Figures S3, S4 and S5 are summarized in Figure 2 of the main article.

S2 Machine Learning Interatomic Potentials

Machine learning interatomic potentials (MLIPs) were developed using the MACE architecture[19] configured with two layers, a spherical expansion up to $L_{\mathrm{max}} = 3$, and correlation order 3 (thus incorporating up to 4-body interactions). The tensor decomposition dimension was set to 128 channels, with a cutoff radius of 6.5 Å. The choice of the cutoff radius was set after some test calculations to ensure that the MLIP correctly accounted for the D3 dispersion-correction contribution. In Figure S5, the dispersion-correction energy term of a water dimer as a function of the distance, rigidly moving one molecule away from the other, is depicted to highlight the negligible effect of this contribution beyond 6.5 Å. The energy value of the D3 correction was as low as 10^{-3} kcal/mol at a distance close to 6.5 Å, indicating the appropriate inclusion within the training data set.

Three MLIP models for water were trained: one incorporating the optimized D3 dispersion corrections (revPBE-D3^{OPT}), one incorporating the default D3(0) dispersion corrections and the other one based on the pristine revPBE functional without corrections. Water models were trained for 120 epochs. The training performance, measured by the root mean square error (RMSE) of energies and forces per epoch, is depicted in Figure S6 for the revPBE-D3^{OPT} model (similar convergence was obtained for the other models, data not shown). The inset in Figure S6 reports a zoomed view of the last 20 epochs and confirms that full convergence was reached with an improvement of just 0.01 meV/atom in energy and 0.15 meV/Å in forces. Two more MLIP models were trained for Mg^{2+} aqueous solutions one using the standard parameterization of the D3(0) for water-water interaction and of the D4 for Mg^{2+}-water and another one using the optimized version of both D3(0) for water-water and D4 for Mg^{2+}-water. The database used for the training contains a subset (containing

only the 128 Water system and centroid configurations for a total of 8333 structures) of the dataset used to train the MLIP-*rev*PBE-D3^{OPT} and the 7865 structures sampled from the Mg-Cl_2 system.

To further validate our choice of the dispersion corrections, we performed Born–Oppenheimer MD (BO-MD) simulations using the reference DFT functional (revPBE) without dispersion corrections. From these simulations, we extracted 13,865 unique structures, which were not included in the original training set, allowing us to evaluate the predictive capability of the obtained MLIP-revPBE-D3^{OPT} model on unseen data. We subsequently applied the D3^{OPT} dispersion correction to these structures to assess how well the MLIP captures dispersion effects not explicitly trained on. In Figure S7, we compare energies and atomic forces computed with both pure DFT and corresponding MLIP, considering results from revPBE and revPBE-D3^{OPT} calculations. The root-mean-square errors (RMSE) for energies were 0.1 meV/atom for both revPBE and revPBE-D3^{OPT}. For the atomic forces, the RMSE was 8.08 meV/Å for revPBE and 8.42 meV/Å for revPBE-D3^{OPT}, indicating excellent consistency and confirming that the MLIPs reliably reproduced both standard DFT and dispersion-corrected interactions. Training time for all models is around 13 hours using 16 A100 GPUs in parallel (roughly 200 GPU hours). When deployed in LAMMPS on a single A100 GPU, a 1500-atom system advances up to 0.7 ns/day in the NpT ensemble and up to 1.2 ns/day in the NVT.

S3 MLIP Database Sampling

To ensure that the MLIP is trained and validated on a diverse and representative set of configurations, we adopted a three-stage sampling workflow with explicit diversity checks. First, liquid-water configurations were generated from classical MD using TIP4P/2005.[20] We saved one frame every 100 ps to enforce temporal decorrelation; every tenth saved frame (i.e., spaced by 1 ns) was pre-relaxed for 20 geometry steps at the reference *rev*PBE–D^{OPT}

level with oxygen positions frozen. Two periodic system sizes (64 and 128 H_2O) were used to mitigate finite-size effects. This initial database comprised 10 200 structures (5 100 with 64 H_2O and 5 100 with 128 H_2O).

An initial model, MLIP$^{(1)}$, trained on the above set was then used to drive additional NpT simulations so that resampling was coherent with the target PES (rather than the TIP4P/2005 PES). From these trajectories, configurations were extracted every 25 ps and appended to the database, yielding a second model, MLIP$^{(2)}$.

To enrich rare but physically relevant regions, MLIP$^{(2)}$ was employed to generate (i) path-integral MD (PIMD) trajectories for bulk water, broadening the distribution of hydrogen-bond geometries (short/long O–H and O$\cdots$O contacts), and (ii) well-tempered metadynamics[21,22] for $MgCl_2$ in water using the Mg–O coordination number (Eq. 10) as a collective variable to enhance sampling of first-shell distortions, under/over-coordination, and water-exchange pathways. Harvested frames from both protocols were added to the database before final training. To further increase diversity we also included cluster structures (water and Mg^{2+}–water) used during the DFT-D optimization.

S4 MD Simulation Details

Classical MD simulation using the TIP4P[20] model, used to start the sampling of water configurations, was carried out in the gromacs[23] using the Nose-Hoover barostat and thermostat to equilibrate the system at 300K and 1 atm in the NpT ensemble. The sampling was then performed in the NVT ensemble using a timestep of 0.5[24] fs. Classical MD simulations of Mg2+ solutions were carried out using two of the most popular Force Fields for magnesium ions (MicroMg[25] and Li-Merz 12-6-4[26]) in Amber22.[27] A single Mg^{2+} ion was solvated in a 64 nm^3 box, using TIP3P water model[28] to model the solvent. We minimized the systems with 20000 steps of steepest descent followed by 10000 steps of conjugate gradient. We performed a 250 ps NpT heating procedure to heat the system from 0 K to 300 K followed by

a 1 ns equilibration at 300K with constant NpT conditions, setting the pressure at 1 atm using Berendsen barostat.[29] We used the equilibrated geometries for 5 ns production runs to obtain the RDFs. An integration time step of 1 fs was used in the heating steps and 2 fs in the production runs. Langevin dynamics temperature[30] control was employed in the heating and production runs with a collision rate equal to 1.0 ps. All simulations with classical force field used the PME[31] algorithm to treat long-range interactions with a 12 Å cutoff.

MD simulations of liquid water using MLIP were performed on a system of 510 water molecules in LAMMPS[32] with a timestep of 0.5 fs.[24] The starting configurations for the various ice phases were obtained by replicating the structures of the DMC-ICE13 dataset twice in each of the periodic directions. To obtain the density isobars presented in Figure 4 of the main article, NpT simulations of 2.5 ns were performed. The Langevin thermostat and barostat were employed with a τ_T of 500 fs and a τ_p of 5 ps. Equilibrium values were evaluated from the last ns of each simulation run. In Figure S8, a comparison between our model and recent studies using MB-(DC)SCAN,[33] BP-revPBE0-D3,[34] and AI-revPBE-D3[35] is reported, to complement Figure 4 of the main article. Structural properties such as radial distribution functions (RDF) (Figure 3 of the main article and Figure S2), hydrogen bond angle distributions (Figure 3), and the tetrahedral structure factor q (Figure S2) were all evaluated from NVT simulations. Dynamic properties such as diffusion coefficients and power spectrum were evaluated from NVE trajectories. In Figure S10 we also report the O-O RDF plotted to 1 nm, demonstrating that the distribution remains continuous at longer distances.

Path-integral (PI) MD simulations of liquid water were carried out using the PIGLET[36] scheme as implemented in i-PI,[37] employing the implementation of MACE in ASE[38] as a driver. We imposed a 56-bead representation to account for quantum nuclear fluctuations with a timestep of 0.25 fs. To reproduce NVT and NpT simulations, we applied a generalized Langevin equation (GLE) thermostat via PIGLET to both the centroid and the internal ring-polymer modes. For NpT simulations, an isotropic barostat with its own Langevin ther-

mostat was additionally employed to maintain the target pressure. For dynamical properties (i.e. Diffusion and Velocity Autocorrelation Function) we sampled the centroid dynamics with TRPMD. All quantum MD simulations were performed on systems of 64 water molecules in a cubic box with a side length of approximately 1.24 nm. Density was evaluated by carrying out PI-MD simulations for about 200 ps. Molecular Representations were produced using VMD.[39]

S5 Metadynamics

Plumed 2.9.0[40,41] was used to drive Well-Tempered Metadynamics[21,22] (WT-MetaD) simulations. For MLIPs we ran our Meta-MD simulations on a pre-equilibrated system containing 127 water molecules and a Magnesium ion. We used NVT dynamics and 4 multiple walkers depositing a gaussian bias, with height 0.96 KJ/mol, every ps.

We also employed WT-MetaD to obtain water coordination profiles of popular classical FF for magnesium ions (MicroMg,[25] 12-6-4[26]). We used the last frame geometries equilibrated as described in Section S3 to perform 50 ns WT-MetaD simulations, depositing a Gaussian bias of height 0.96 KJ/mol every ps.

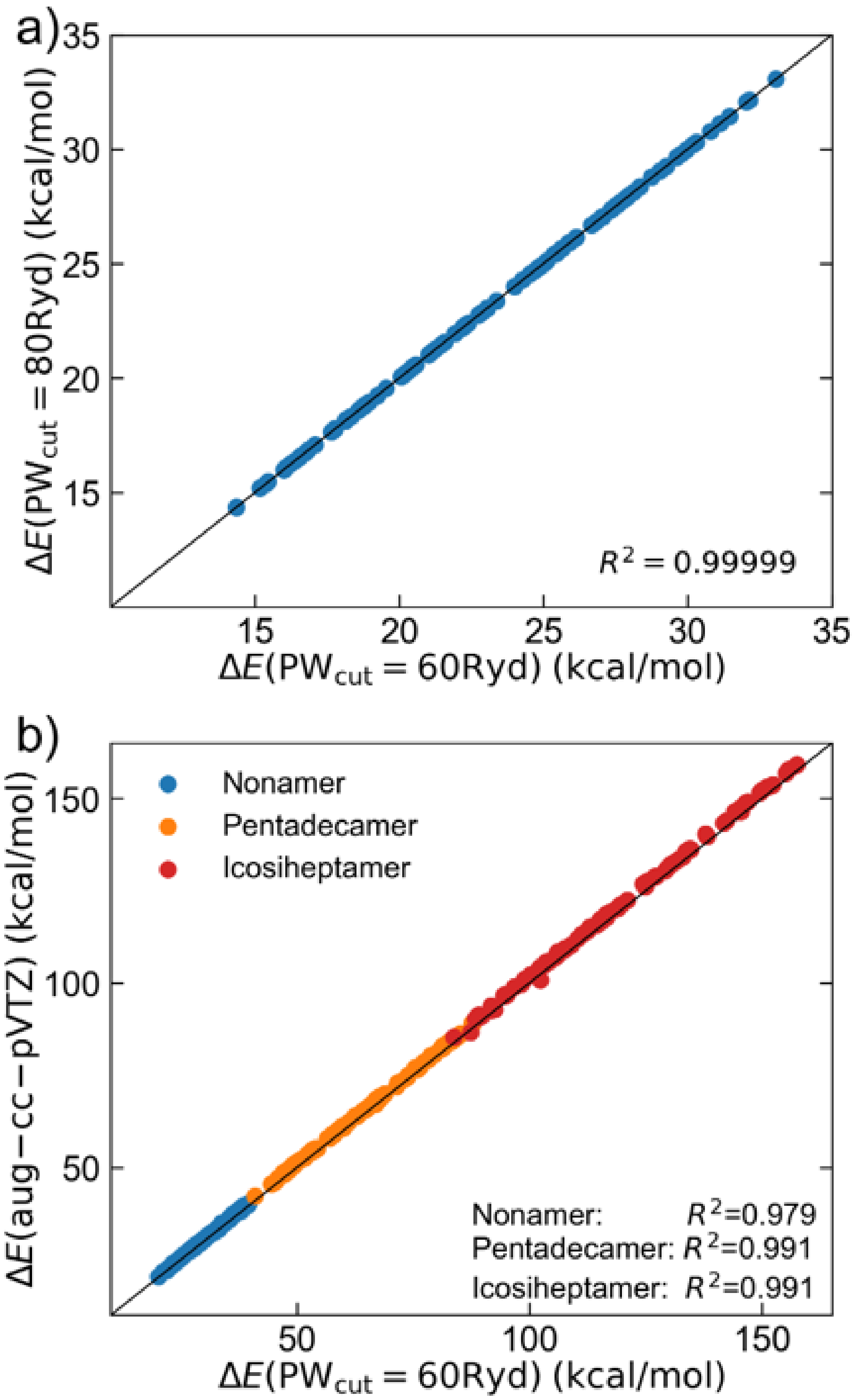

Figure S1: (a) Interaction Energy comparison between 60 and 80 Ryd cutoff on plane-wave energy on 100 9-mer structures. (b) Interaction Energy comparison between plane waves and atom-centered Gaussian orbitals (aug-cc-pVTZ) on 300 cluster structures (9mer, 15mer, and 27mer).

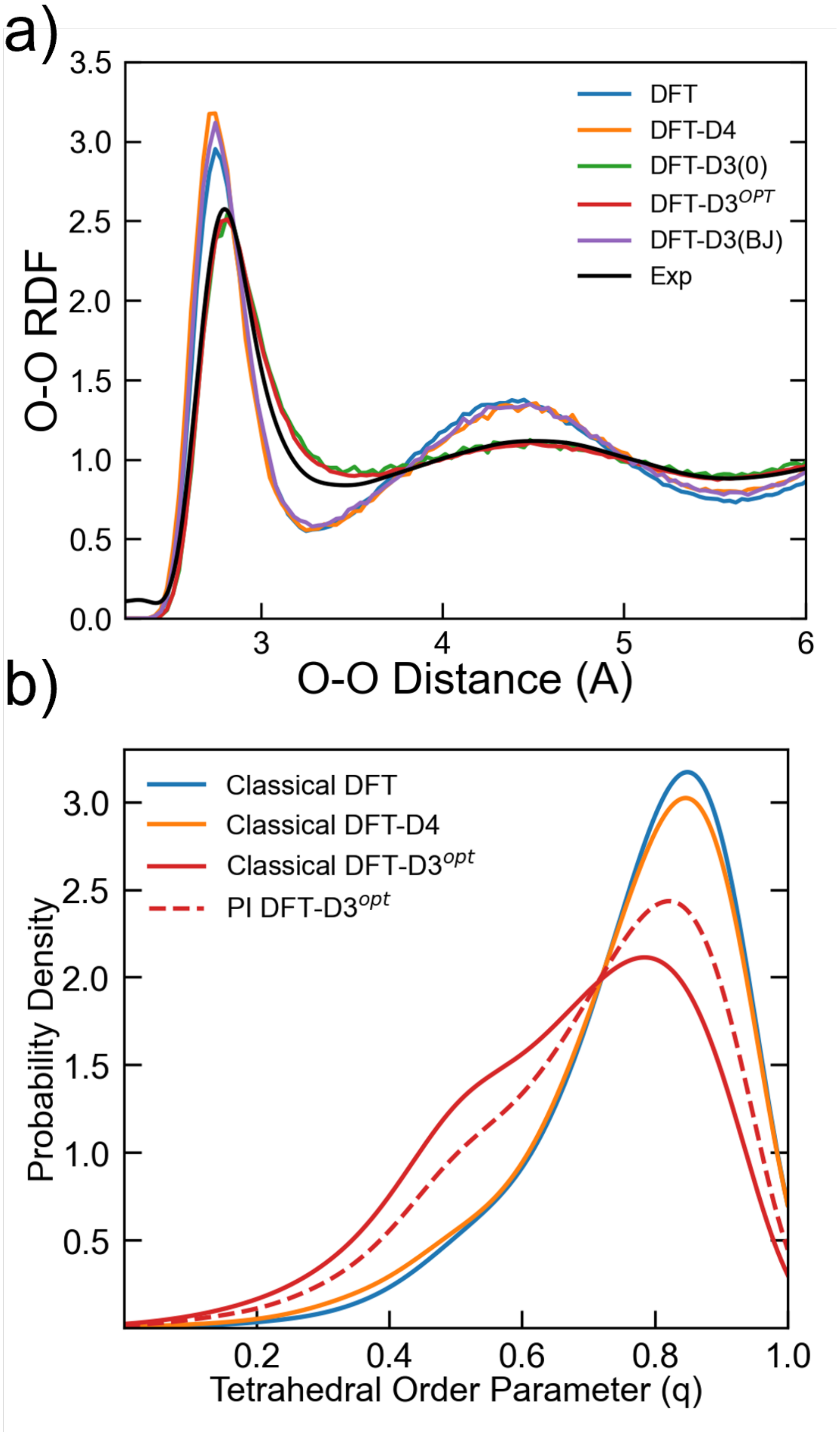

Figure S2: (a) O-O radial distribution function from liquid water simulations at normal conditions evaluated using revPBE (DFT) combined with (or without) different dispersion-correction models. Experimental reference was replotted from Skinner et al.[42] (b) Tetrahedral structure factor q evaluated using revPBE (DFT) combined with (or without) different dispersion-correction models.

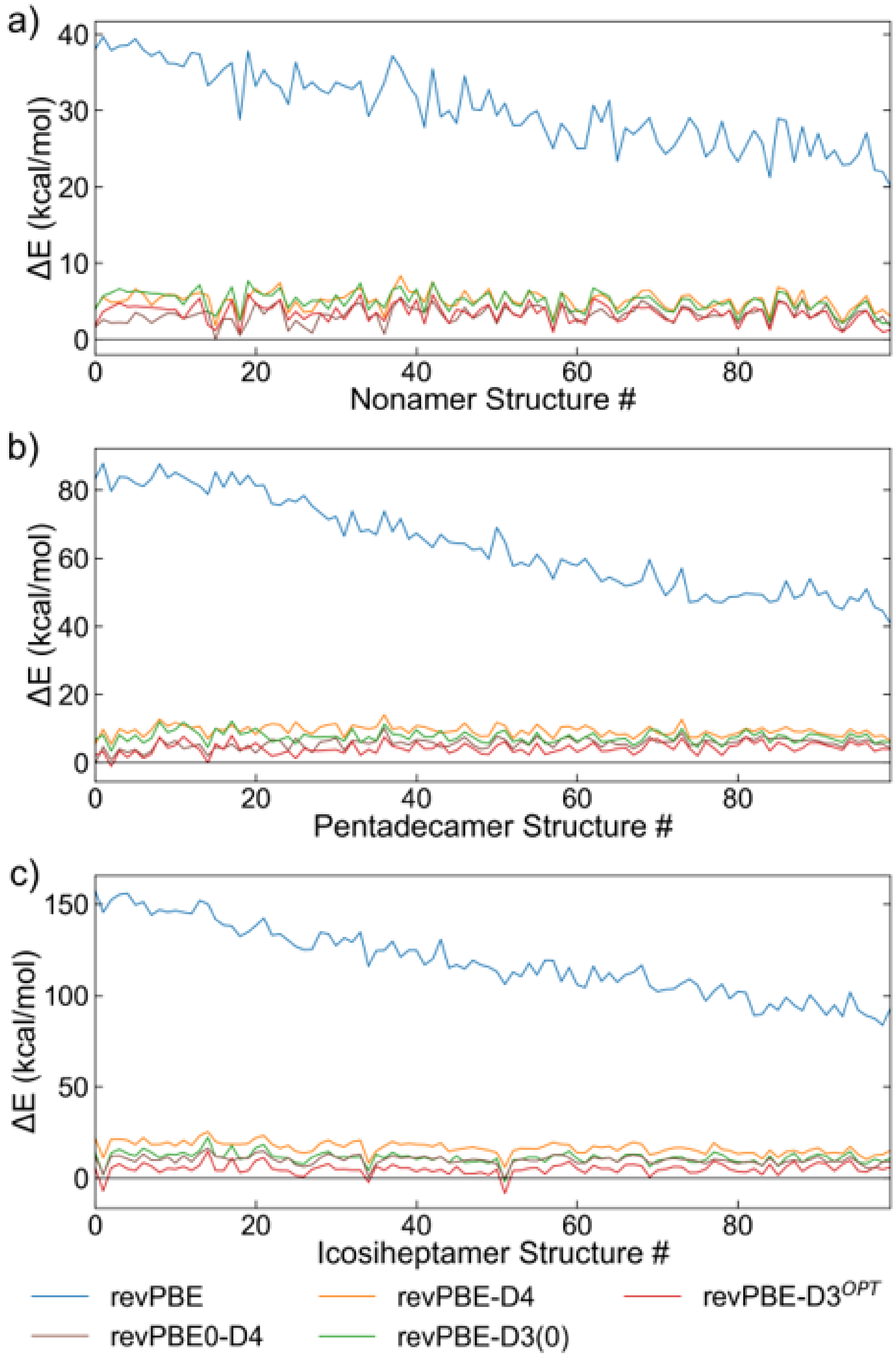

Figure S3: Interaction energy deviations with respect to DMC evaluated on 300 water cluster structures: a) 9mer cluster (100 structures), b) 15mer cluster (100 structures), and c) 27mer cluster (100 structures). DFT calculations were carried out using plane-wave basis set with 60 Ryd cutoff on energy and 300 Ryd on density. revPBE0-D4 results were taken from ref.[15] using aug-cc-pVTZ basis set.

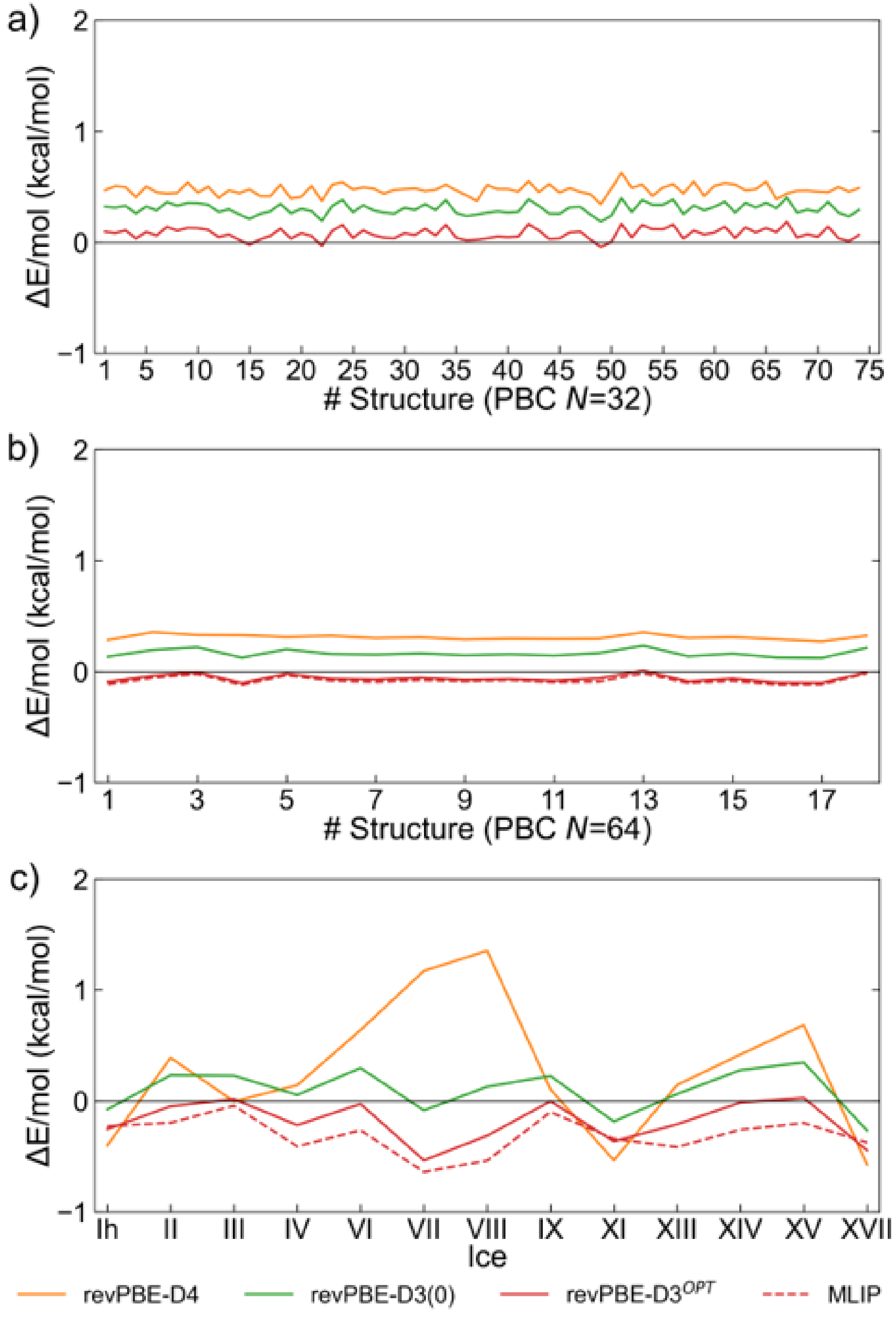

Figure S4: Interaction energy deviations with respect to DMC on periodic structures of liquid water a) 32 water molecule system (75 structures) and b) 64 water molecule system (18 structures) and c) various polymorphic phases of ice from the DMC-ICE13 dataset.[17] DFT calculations were carried out using plane-wave basis set with 60 Ryd cutoff on energy and 300 Ryd on density. MLIP results are issuing from the MLIP-revPBE-D3^{OPT} model.

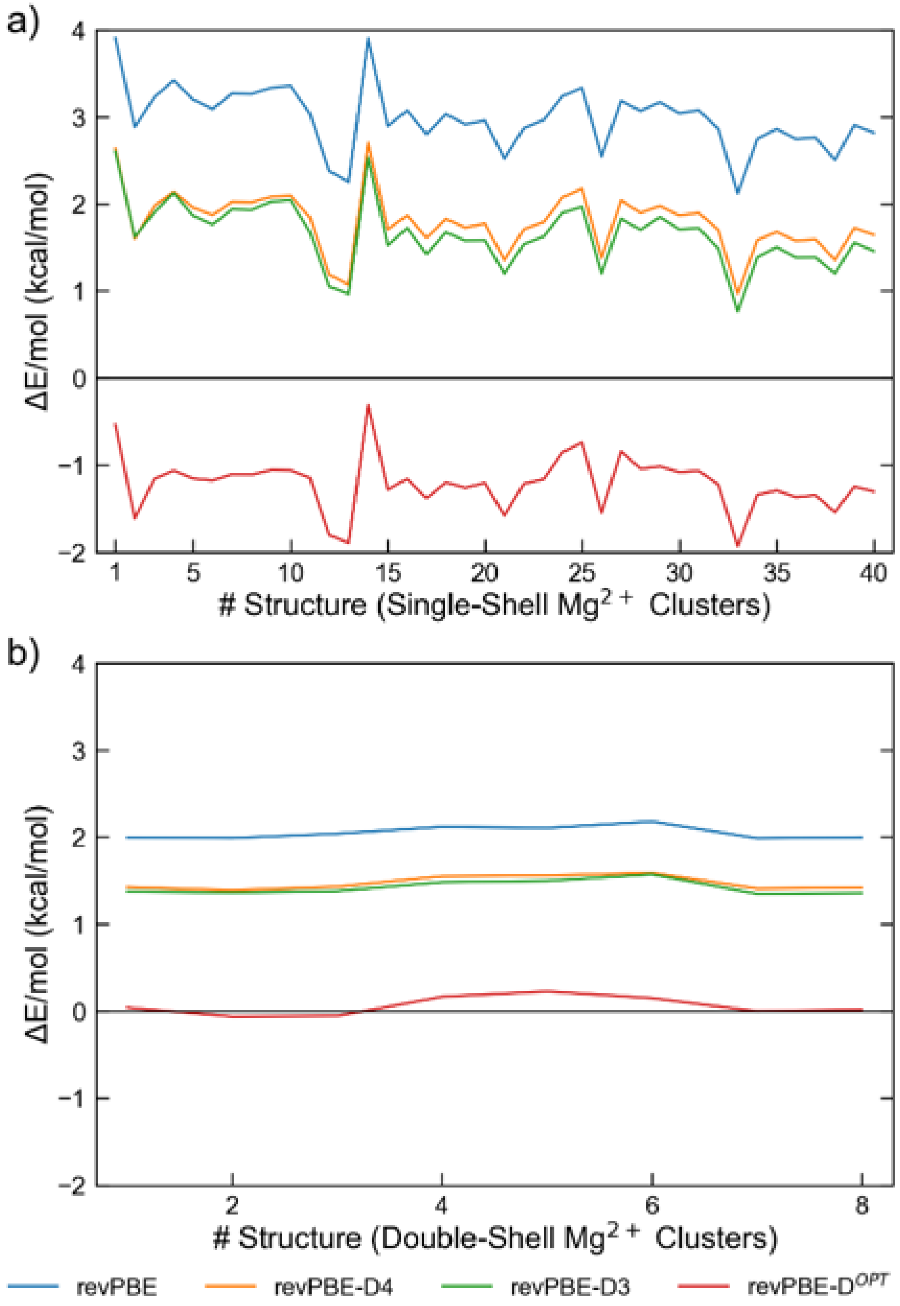

Figure S5: Interaction energy deviations with respect to DLPNO-CCSD(T)-F12 on Mg^{2+}-water clusters a) Single-shell systems (40 structure) and b) Double-shell system (8 structures). DFT calculations were carried out using plane-wave basis set with 60 Ryd cutoff on energy and 300 Ryd on density.

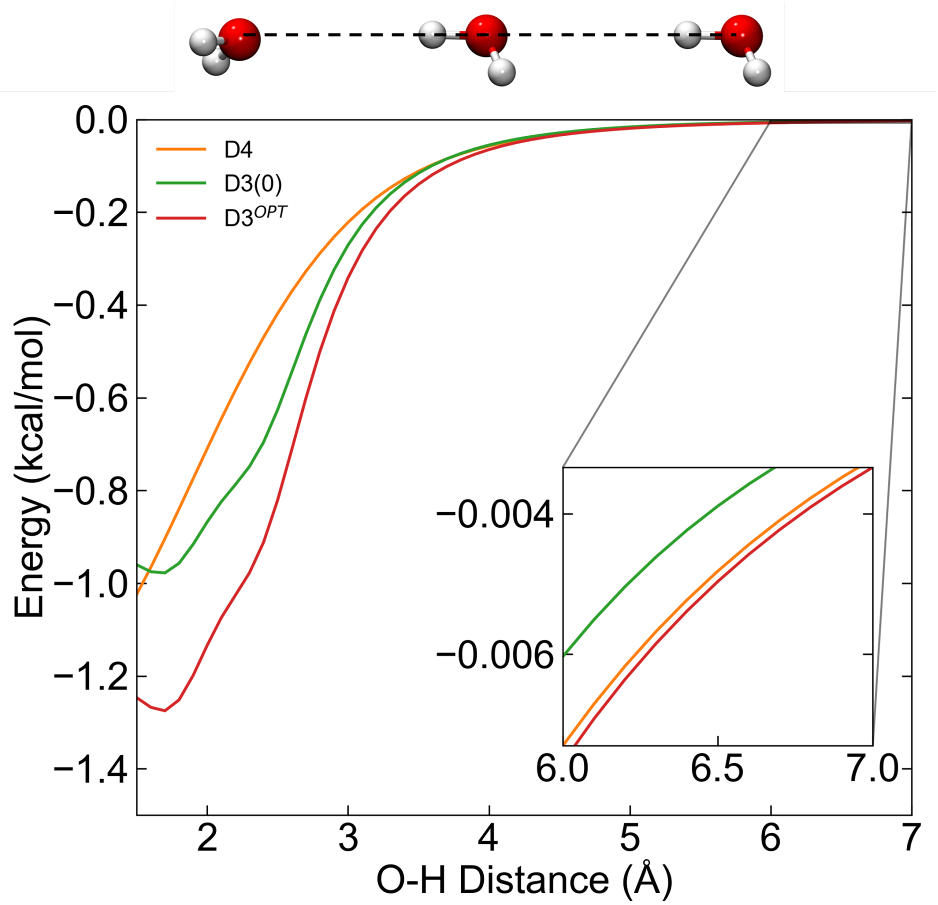

Figure S6: Dispersion-correction energy of a water dimer, rigidly moving one molecule form the other along the direction of an hydrogen bond.

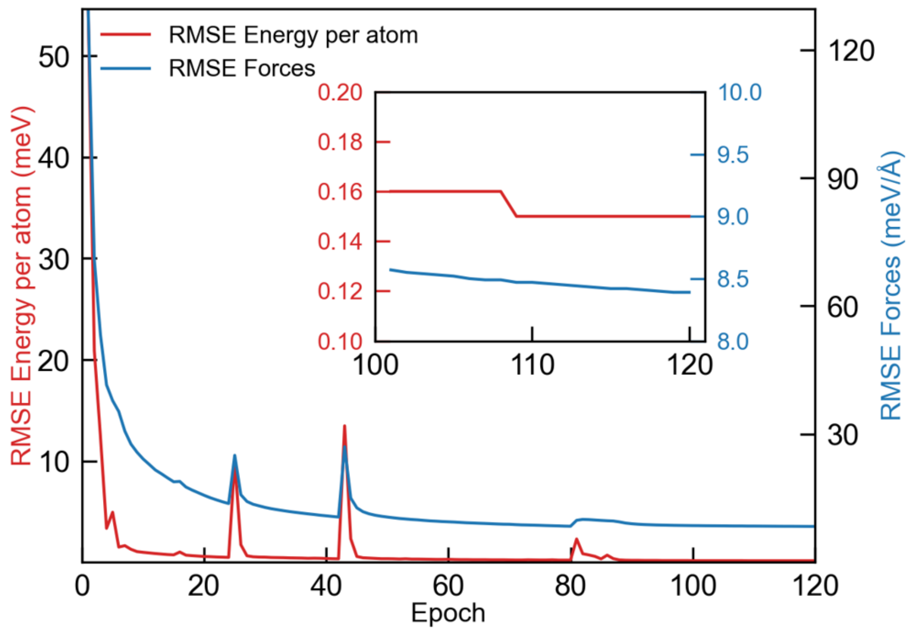

Figure S7: RMSE of energy per atom and forces as a function of the epoch during MLIP training on revPBE-D3^{OPT} data. Inset, zoom view of the last 20 epochs.

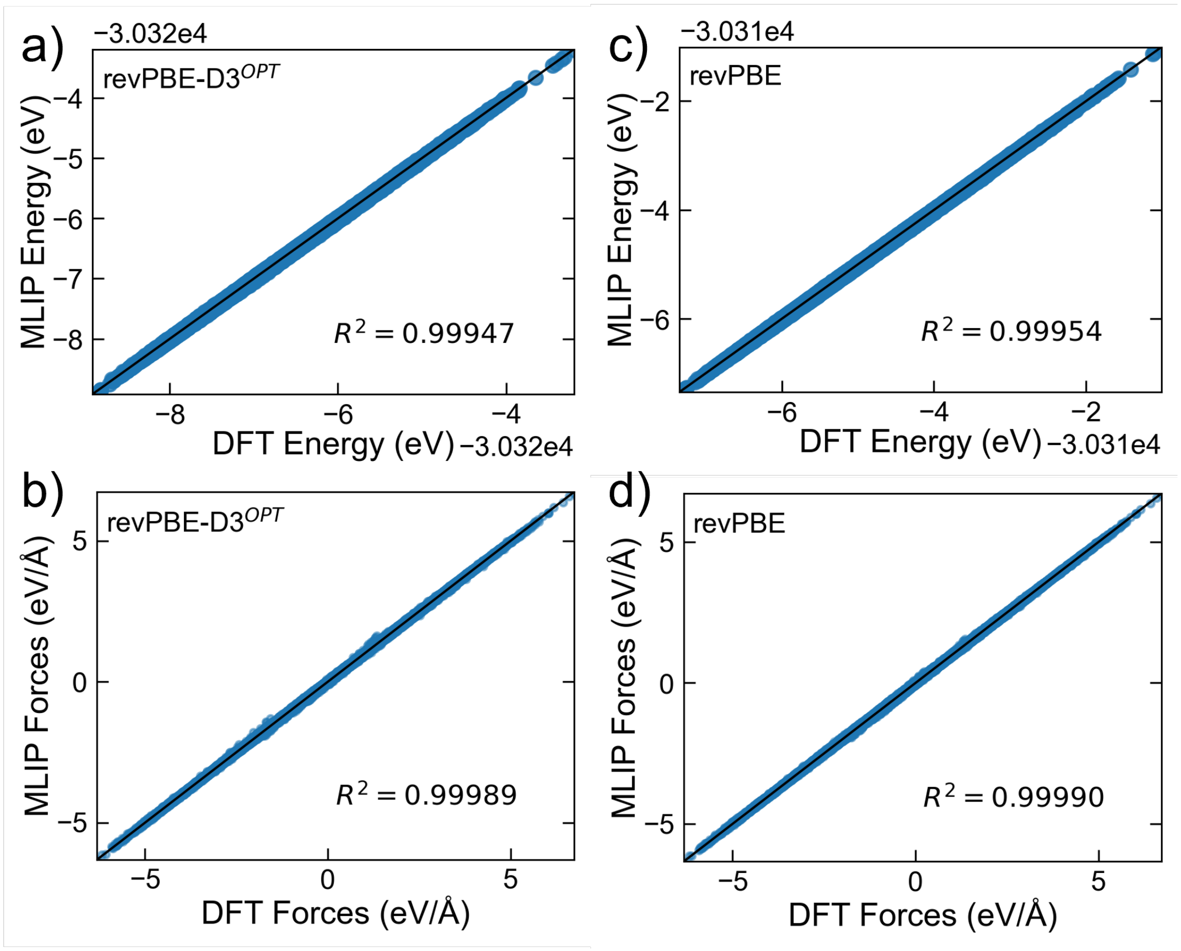

Figure S8: **a-d):** Comparison of energies and atomic forces between DFT (revPBE or revPBE-D3^{OPT}) and corresponding MLIP on a dataset of 13865 structures from a Born-Oppenheimer MD trajectory. RMSE E 0.1 meV/atom for both models, RMSE F 8.08 meV/Å and 8.42 mev/Å for revPBE revPBE-D3^{OPT} and respectively.

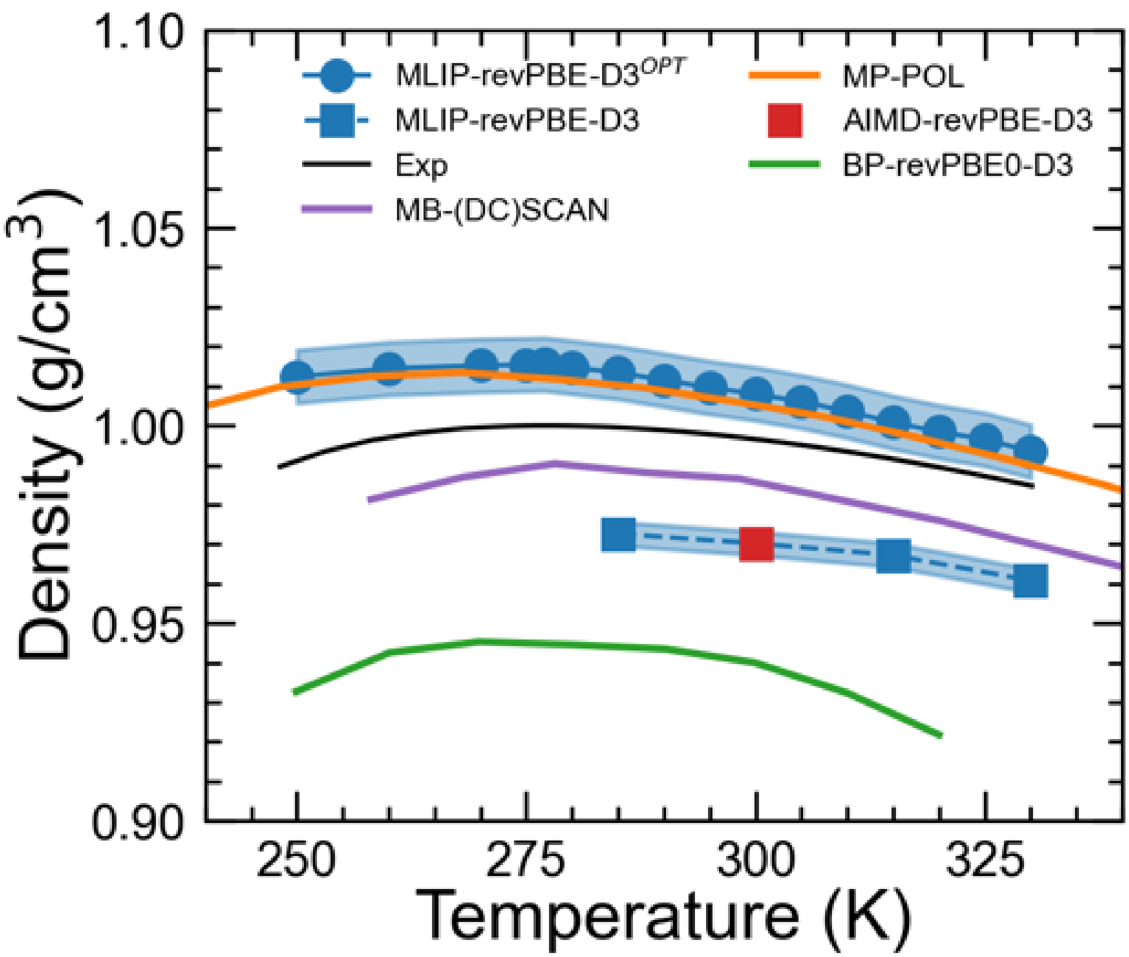

Figure S9: Experimental and simulated density isobars obtained using the MLIP-revPBE-D3^{OPT} and MLIP-*rev*PBE-D3 models from this work, Belher-Parinnello revPBE0-D3 (extracted and replotted from ref.[34]), MB-(DC)SCAN (extracted and replotted from ref.[33]), MB-POL (extracted and replotted from ref.[43] and AIMD rev-PBE-D3 from ref.[35] Note how the latter *ab initio* result matched very well the results from our MLIP-*rev*PBE-D3 model, thus supporting the present MLIP implementation. Experimental densities were replotted from NIST.[44]

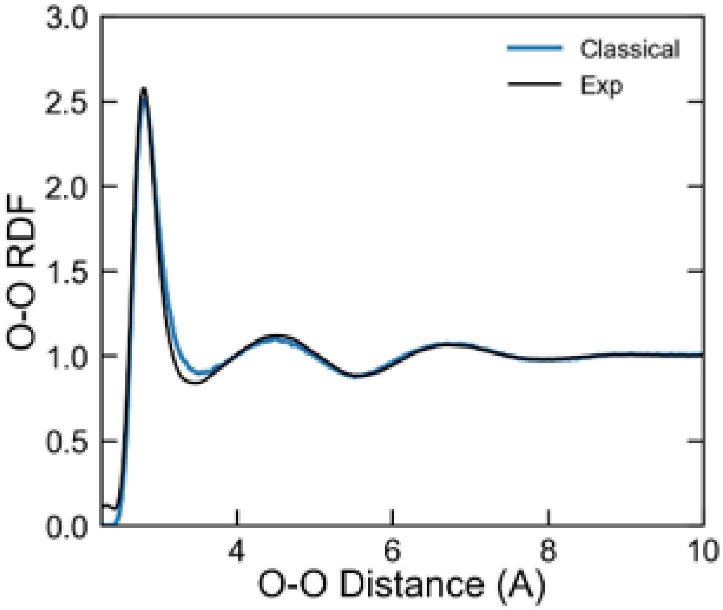

Figure S10: O-O RDF from classical simulations using the MLIP-*rev*PBE-D3^{OPT} on longer distances. Experiment was replotted from Skinner et al.[42]

References

(1) Giannozzi, P. et al. QUANTUM ESPRESSO: a modular and open-source software project for quantum simulations of materials. *Journal of Physics: Condensed Matter* **2009**, *21*, 395502.

(2) Zhang, Y.; Yang, W. Comment on "Generalized Gradient Approximation Made Simple". *Physical Review Letters* **1998**, *80*, 890–890, Publisher: American Physical Society.

(3) Dal Corso, A. Pseudopotentials periodic table: From H to Pu. *Computational Materials Science* **2014**, *95*, 337–350.

(4) Dunning, T. H., Jr. Gaussian basis sets for use in correlated molecular calculations. I. The atoms boron through neon and hydrogen. *The Journal of Chemical Physics* **1989**, *90*, 1007–1023.

(5) Grimme, S.; Antony, J.; Ehrlich, S.; Krieg, H. A consistent and accurate ab initio parametrization of density functional dispersion correction (DFT-D) for the 94 elements H-Pu. *The Journal of Chemical Physics* **2010**, *132*, 154104.

(6) Caldeweyher, E.; Bannwarth, C.; Grimme, S. Extension of the D3 dispersion coefficient model. *The Journal of Chemical Physics* **2017**, *147*, 034112.

(7) Caldeweyher, E.; Ehlert, S.; Hansen, A.; Neugebauer, H.; Spicher, S.; Bannwarth, C.; Grimme, S. A generally applicable atomic-charge dependent London dispersion correction. *The Journal of Chemical Physics* **2019**, *150*, 154122.

(8) Caldeweyher, E.; Mewes, J.-M.; Ehlert, S.; Grimme, S. Extension and evaluation of the D4 London-dispersion model for periodic systems. *Physical Chemistry Chemical Physics* **2020**, *22*, 8499–8512, Publisher: The Royal Society of Chemistry.

(9) Neese, F. The ORCA program system. *WIREs Computational Molecular Science* **2012**, *2*, 73–78, _eprint: https://wires.onlinelibrary.wiley.com/doi/pdf/10.1002/wcms.81.

(10) Grimme, S.; Ehrlich, S.; Goerigk, L. Effect of the damping function in dispersion corrected density functional theory. *Journal of Computational Chemistry* **2011**, *32*, 1456–1465, _eprint: https://onlinelibrary.wiley.com/doi/pdf/10.1002/jcc.21759.

(11) Wang, J.; Román-Pérez, G.; Soler, J. M.; Artacho, E.; Fernández-Serra, M.-V. Density, structure, and dynamics of water: The effect of van der Waals interactions. *The Journal of Chemical Physics* **2011**, *134*, 024516.

(12) Lausch, K. N.; El Haouari, R.; Trzewik, D.; Behler, J. Impact of the damping function in dispersion-corrected density functional theory on the properties of liquid water. *The Journal of Chemical Physics* **2025**, *163*, 034101.

(13) Alfè, D.; Bartók, A. P.; Csányi, G.; Gillan, M. J. Communication: Energy benchmarking with quantum Monte Carlo for water nano-droplets and bulk liquid water. *The Journal of Chemical Physics* **2013**, *138*, 221102, Publisher: American Institute of Physics.

(14) Barbosa, N.; Pagliai, M.; Sinha, S.; Barone, V.; Alfè, D.; Brancato, G. Enhancing the Accuracy of Ab Initio Molecular Dynamics by Fine Tuning of Effective Two-Body Interactions: Acetonitrile as a Test Case. *The Journal of Physical Chemistry A* **2021**, *125*, 10475–10484, Publisher: American Chemical Society.

(15) Ferretti, A.; Canal, L.; Sorodoc, R. A.; Sinha, S.; Brancato, G. Fine Tuning the Intermolecular Interactions of Water Clusters Using the Dispersion-Corrected Density Functional Theory. *Molecules* **2023**, *28*, 3834, Number: 9 Publisher: Multidisciplinary Digital Publishing Institute.

(16) Alfè, D.; Bartók, A. P.; Csányi, G.; Gillan, M. J. Analyzing the errors of DFT approximations for compressed water systems. *The Journal of Chemical Physics* **2014**, *141*, 014104, Publisher: American Institute of Physics.

(17) Della Pia, F.; Zen, A.; Alfè, D.; Michaelides, A. DMC-ICE13: Ambient and high pressure polymorphs of ice from diffusion Monte Carlo and density functional theory. *The Journal of Chemical Physics* **2022**, *157*, 134701.

(18) Adamo, C.; Barone, V. Toward reliable density functional methods without adjustable parameters: The PBE0 model. *The Journal of Chemical Physics* **1999**, *110*, 6158–6170.

(19) Batatia, I.; Kovács, D. P.; Simm, G. N. C.; Ortner, C.; Csányi, G. MACE: Higher Order Equivariant Message Passing Neural Networks for Fast and Accurate Force Fields. 2023; `http://arxiv.org/abs/2206.07697`, arXiv:2206.07697 [cond-mat, physics:physics, stat].

(20) Abascal, J. L. F.; Vega, C. A general purpose model for the condensed phases of water: TIP4P/2005. *The Journal of Chemical Physics* **2005**, *123*, 234505.

(21) Laio, A.; Parrinello, M. Escaping free-energy minima. *Proceedings of the National Academy of Sciences* **2002**, *99*, 12562–12566, Publisher: Proceedings of the National Academy of Sciences.

(22) Barducci, A.; Bussi, G.; Parrinello, M. Well-Tempered Metadynamics: A Smoothly Converging and Tunable Free-Energy Method. *Physical Review Letters* **2008**, *100*, 020603, Publisher: American Physical Society.

(23) Abraham, M. J.; Murtola, T.; Schulz, R.; Páll, S.; Smith, J. C.; Hess, B.; Lindahl, E. GROMACS: High performance molecular simulations through multi-level parallelism from laptops to supercomputers. *SoftwareX* **2015**, *1-2*, 19–25.

(24) Asthagiri, D. N.; Beck, T. L. MD Simulation of Water Using a Rigid Body Description Requires a Small Time Step to Ensure Equipartition. *Journal of Chemical Theory and Computation* **2024**, *20*, 368–374, Publisher: American Chemical Society.

(25) Grotz, K. K.; Cruz-León, S.; Schwierz, N. Optimized Magnesium Force Field Parameters for Biomolecular Simulations with Accurate Solvation, Ion-Binding, and Water-Exchange Properties. *Journal of Chemical Theory and Computation* **2021**, *17*, 2530–2540, Publisher: American Chemical Society.

(26) Li, P.; Song, L. F.; Merz, K. M. J. Parameterization of Highly Charged Metal Ions Using the 12-6-4 LJ-Type Nonbonded Model in Explicit Water. *The Journal of Physical Chemistry B* **2015**, *119*, 883–895, Publisher: American Chemical Society.

(27) Case, D. A.; Duke, R. E.; Walker, R. C.; Skrynnikov, N. R.; Cheatham III, T. E.; Mikhailovskii, O.; Simmerling, C.; Xue, Y.; Roitberg, A.; Izmailov, S. A.; others AMBER 22 reference manual. **2022**,

(28) Jorgensen, W. L.; Chandrasekhar, J.; Madura, J. D.; Impey, R. W.; Klein, M. L. Comparison of simple potential functions for simulating liquid water. *The Journal of Chemical Physics* **1983**, *79*, 926–935.

(29) Berendsen, H. J. C.; Postma, J. P. M.; van Gunsteren, W. F.; DiNola, A.; Haak, J. R. Molecular dynamics with coupling to an external bath. *The Journal of Chemical Physics* **1984**, *81*, 3684–3690.

(30) Loncharich, R. J.; Brooks, B. R.; Pastor, R. W. Langevin dynamics of peptides: The frictional dependence of isomerization rates of N-acetylalanyl-N'-methylamide. *Biopolymers* **1992**, *32*, 523–535.

(31) Essmann, U.; Perera, L.; Berkowitz, M. L.; Darden, T.; Lee, H.; Pedersen, L. G. A smooth particle mesh Ewald method. *The Journal of Chemical Physics* **1995**, *103*, 8577–8593.

(32) Thompson, A. P.; Aktulga, H. M.; Berger, R.; Bolintineanu, D. S.; Brown, W. M.; Crozier, P. S.; in 't Veld, P. J.; Kohlmeyer, A.; Moore, S. G.; Nguyen, T. D.; Shan, R.;

Stevens, M. J.; Tranchida, J.; Trott, C.; Plimpton, S. J. LAMMPS - a flexible simulation tool for particle-based materials modeling at the atomic, meso, and continuum scales. *Computer Physics Communications* **2022**, *271*, 108171.

(33) Dasgupta, S.; Lambros, E.; Perdew, J. P.; Paesani, F. Elevating density functional theory to chemical accuracy for water simulations through a density-corrected many-body formalism. *Nature Communications* **2021**, *12*, 6359, Publisher: Nature Publishing Group.

(34) Cheng, B.; Engel, E. A.; Behler, J.; Dellago, C.; Ceriotti, M. Ab initio thermodynamics of liquid and solid water. *Proceedings of the National Academy of Sciences* **2019**, *116*, 1110–1115, Publisher: Proceedings of the National Academy of Sciences.

(35) Pestana, L. R.; Mardirossian, N.; Head-Gordon, M.; Head-Gordon, T. Ab initio molecular dynamics simulations of liquid water using high quality meta-GGA functionals. *Chemical Science* **2017**, *8*, 3554–3565, Publisher: The Royal Society of Chemistry.

(36) Uhl, F.; Marx, D.; Ceriotti, M. Accelerated path integral methods for atomistic simulations at ultra-low temperatures. *The Journal of Chemical Physics* **2016**, *145*, 054101.

(37) Kapil, V. et al. i-PI 2.0: A universal force engine for advanced molecular simulations. *Computer Physics Communications* **2019**, *236*, 214–223.

(38) Hjorth Larsen, A. et al. The atomic simulation environment—a Python library for working with atoms. *Journal of Physics: Condensed Matter* **2017**, *29*, 273002, Publisher: IOP Publishing.

(39) Humphrey, W.; Dalke, A.; Schulten, K. VMD: Visual molecular dynamics. *Journal of Molecular Graphics* **1996**, *14*, 33–38.

(40) Tribello, G. A.; Bonomi, M.; Branduardi, D.; Camilloni, C.; Bussi, G. PLUMED 2: New feathers for an old bird. *Computer Physics Communications* **2014**, *185*, 604–613.

(41) Bonomi, M. et al. Promoting transparency and reproducibility in enhanced molecular simulations. *Nature Methods* **2019**, *16*, 670–673, Publisher: Nature Publishing Group.

(42) Skinner, L. B.; Galib, M.; Fulton, J. L.; Mundy, C. J.; Parise, J. B.; Pham, V.-T.; Schenter, G. K.; Benmore, C. J. The structure of liquid water up to 360 MPa from x-ray diffraction measurements using a high Q-range and from molecular simulation. *The Journal of Chemical Physics* **2016**, *144*, 134504.

(43) Zhu, X.; Riera, M.; Bull-Vulpe, E. F.; Paesani, F. MB-pol(2023): Sub-chemical Accuracy for Water Simulations from the Gas to the Liquid Phase. *Journal of Chemical Theory and Computation* **2023**, *19*, 3551–3566, Publisher: American Chemical Society.

(44) Harvey, A. H. Properties of Ice and Supercooled Water. *NIST* **2019**, Last Modified: 2020-09-28T11:09-04:00 Publisher: Allan H. Harvey.
